# Distributed Target Tracking in Challenging Environments Using Multiple Asynchronous Bearing-Only Sensors

**DOI:** 10.3390/s20092671

**Published:** 2020-05-07

**Authors:** Yifang Shi, Jee Woong Choi, Lei Xu, Hyung June Kim, Ihsan Ullah, Uzair Khan

**Affiliations:** 1 School of Automation, Hangzhou Dianzi University, Xiasha Higher Education Zone, 2rd Street, Hangzhou 310018, China; syf2008@hdu.edu.cn; 2 Department of Marine Science and Convergence Engineering, Hanyang University, Ansan 15588, Korea; choijw@hanyang.ac.kr; 3 R&D Center, Shenzhen XuanQi Intelligent Technology Co., Ltd., Shenzhen 518000, China; xuleialfred@gmail.com; 4 Department of Electronic Systems Engineering, Hanyang University, Ansan 15588, Korea; lovesunday@hanyang.ac.kr; 5 Department of Electrical Engineering, COMSATS University, Abbottabad Campus, Abbottabad 22060, Pakistan; uzairkhan@ciit.net.pk

**Keywords:** multiple asynchronous BO sensors tracking, track management, OOS information, distributed tracking, LIPDA, DIPDA-FPFD

## Abstract

In the multiple asynchronous bearing-only (BO) sensors tracking system, there usually exist two main challenges: (1) the presence of clutter measurements and the target misdetection due to imperfect sensing; (2) the out-of-sequence (OOS) arrival of locally transmitted information due to diverse sensor sampling interval or internal processing time or uncertain communication delay. This paper simultaneously addresses the two problems by proposing a novel distributed tracking architecture consisting of the local tracking and central fusion. To get rid of the kinematic state unobservability problem in local tracking for a single BO sensor scenario, we propose a novel local integrated probabilistic data association (LIPDA) method for target measurement state tracking. The proposed approach enables eliminating most of the clutter measurement disturbance with increased target measurement accuracy. In the central tracking, the fusion center uses the proposed distributed IPDA-forward prediction fusion and decorrelation (DIPDA-FPFD) approach to sequentially fuse the OOS information transmitted by each BO sensor. The track management is carried out at local sensor level and also at the fusion center by using the recursively calculated probability of target existence as a track quality measure. The efficiency of the proposed methodology was validated by intensive numerical experiments.

## 1. Introduction

Target tracking uses noisy observations received by sensors at discrete time instances to sequentially estimate the target state of interest evolving over time. A passive bearing-only (BO) sensor system is able to track a target in a stealthy manner combined with the superior estimation accuracy and low cost. These advantages made it useful in a wide range of military and civilian applications. These applications include but are not limited to control and navigation, surveillance, Internet of Things, just name a few [1,2]. Usually, the data collected from multiple BO sensors are integrated to give a much more accurate and comprehensive description of the targets of interest compared to that of single BO sensor configuration, also with the additional benefit of tackling the target non-observability problem [1].

Compared to centralized tracking architecture, the distributed tracking framework carries out the local tracking and information fusion in any member node of the multisensor system, and has the potential of application to large scale sensor networks, besides, it consumes less computation resources and communication bandwidth, while delivers comparable tracking performance to that of the centralized framework [1,3]. In this paper, target tracking using multiple BO sensors is implemented in a distributed architecture. In realistic multisensor target tracking system, one usually faces two main challenges. The first one is the presence of clutter measurement and target misdetection where the sensor measurements received at each scan contain detections originated not only from targets of interest, but also from thermal noise, terrain reflections, clouds, birds, etc. [4]. Such unwanted measurements are usually termed as clutter measurement disturbance and their number at each scan varies randomly. Both target-originated and clutter-originated measurements simultaneously exist at the measurement space and lead to the measurement origin uncertainty. To make matters worse, even when there are targets in the sensor’s field of view, they can go undetected due to targets occlusion or sensor jamming, resulting in the target misdetection [4]. The problems of clutter measurement disturbance and target misdetection collectively make it considerably difficult to robustly maintain the true tracks and estimate the states of the targets of interest. The second challenge is that the data transmitted from different local sensors unavoidably arrives in the fusion center in out-of-sequence (OOS), i.e., the data measured at earlier time arrives at the fusion center after the central tracks were already updated at the current time. This phenomenon is termed as the OOS information problem and usually happens in the realistic tracking system because of the diverse sensor sampling interval, varying measurement processing time and uncertain communication delay [5,6]. Consequently, updating the currently filtered track state with OOS information becomes nontrivial.

Much research paid attention to the clutter measurement disturbance and target misdetection problem. The authors in [7] first proposed a M/N logic track management methodology, which declares an initialized track to be true track if there are at least M scan gating successes among consecutive N scan gating procedure, while its track management performance may drastically deteriorate as the clutter measurement density increases and the target misdetection exacerbates. Later, the authors in [8,9] proposed using the sequential probability ratio test (SPRT) as the track quality measure to dynamically declare true tracks following targets of interest and recognize false tracks not following any targets of interest. However, the SPRT of each track can be any positive value and determining the track scoring threshold becomes a hard nut. [10] investigated adopting the probability of target visibility to score each tentative track, so as to distinguish true tracks from false tracks. The authors in [11,12] presented a recursively calculated probability of target existence (PTE) as a track quality measure for operating the track management, i.e., confirming true tracks and maintaining them since confirmation, recognizing false tracks and deleting them from memory. In addition to the fact that the PTE is a probability whose value lies between 0 and 1, it is very convenient to set the thresholding value. This shows a prominent improvement in track management [12,13]. The concept of PTE was further extended to deal with the target tracking in the situation of high clutter measurement density and high target misdetection probability by employing a multiple scan data association strategy in [14]. Recently, the authors in [15,16,17,18] introduced a shadowing filter as well as its varieties for target positioning and tracking. In contrast to the sequential tracking methods, the shadowing filters are developed based on a very simple principle: if the model is good enough, state estimations must be close to the observations and consistent with the model’s equations, which imposes a quadratic norm on the filter and guarantees not falling into the trap of local minimum. The availability of the proposed shadowing filters is verified in various tracking applications using real data, which shows novelty and efficiency over the widely used Kalman and particle filters.

The OOS information problem was pervasively studied previously. [19] suggests a straightforward solution by ignoring and directly discarding the OOS information in the tracking procedure, obviously, the useful target information contained in the OOS data are lost. To avoid this drawback, the authors in [20] proposed a reprocessing method which stores all the information collected from the OOS information time to the last track update time and then reprocesses them in a chronological order. This solution gives the optimal tracking performance at the cost of high computation and storage consumption, which is usually not feasible in most of the tracking system. [20,21] investigated an approximated OOS fusion method using the criteria of minimum mean square error under the constraint that only the most recent updates are saved. [22] proposed a fixed-point smoothing based OOS measurement (OOSM) methodology which delivers optimal tracking performance based on the best linear unbiased estimation (BLUE) principle; however, it requires additional storage apart from the state estimate and its associated error covariance. [23] proposed an optimal retrodiction-based OOSM filtering approach termed as A1 algorithm, and also its suboptimal but computationally efficient version called B1 algorithm with less storage requirement, whereas, both A1 and B1 algorithm assumed the OOS lag is less than a sampling interval. The authors in [24] introduced the first optimal solution for the general l-step-lag problem, called the fading information methodology, which updates the current target state using evaluated information from the OOSM on its subsequent states, but it is computationally complex. [25] proposed an augmented state Kalman filter (AS-KF) Bayesian approach to address the OOSM problem, which augments a sequence of recent state to the current state and carry out a batch-form updating strategy, whereas, it needs to approximate the OOSM time to some integer sampling time instance. The authors in [26] presented a single-step retrodiction-based solution for solving l-step-lag OOSM problem termed as the Al1 algorithm and also its computation and storage efficient version Bl1 algorithm. It achieves one-step solution by defining equivalent measurements at the current time that represents all the measurements with time stamps later than the OOSM, and show some priority in the sense of tracking performance and storage requirements among the above reviewed methods. More recently, the authors in [27] enhanced the Al1 and Bl1 algorithms by employing the RTS fixed lag smoothing approach for further improving the fusion performance using Infrared sensor and Laser Detection- Ranging sensor. [28] suggested a new methodology termed as the forward prediction fusion and decorrelation (FPFD) for tackling the OOSM problem without relying on the retrodiction technique, wherein, a tracklet is created and predicted forward and decorrelated from the actual track in the information space. It was proved in [28] that the FPFD method performs as well as the retrodiction-based approaches, while requiring less data storage in most case. The authors in [29] further extended the FPFD concept to tackling the OOS tracks’ fusion problem, and show its potential to be implemented in the real tracking system. While to the best of our knowledge, the above reviewed methodologies all assume an ideal tracking environments and neglect the track management problem in the presence of clutter measurement and target misdetection.

Inspired by enriching the existing work on the multiple BO asynchronous sensors tracking in realistic environments, this paper simultaneously considers the clutter measurement disturbance, target misdetection and the OOS information update problem in the multiple BO sensors tracking system. Within the framework of distributed fusion architecture, the proposed approach consists of a single sensor local tracking and the central fusion. After receiving sets of raw measurements, the local sensor carried out the local pseudo tracking using the proposed local integrated probabilistic data association (LIPDA) method, which tracks the measurement state rather than the target kinematic state (since target kinematic state is unobservable by a single BO sensor). Such a design enables eliminating most of the false tracks via the track management using the recursively computed PTE, resulting in tangibly reduced communication bandwidth and computation complexity, furthermore, the accuracy of target measurement can also be further improved. In the fusion center, we propose to use the distributed integrated probabilistic data association-forward prediction fusion and decorrelation (DIPDA-FPFD) method to sequentially update the current time-filtered track kinematic state using the OOS pseudo track information. Unlike the retrodiction-based methods that need to account for the complicated retrodiction noise, the proposed DIPDA-FPFD algorithm efficiently rules out the dependency between the central track hybrid state updated using OOS information and currently filtered central track hybrid state by employing a straightforward decorrelation technique in the information space. The track management procedure is also carried out in the fusion center to further exclude false tracks and also output target true tracks.

The rest of the paper is organized as follows: the problem statement is described in Section 2, and Section 3 gives an overview of the IPDA algorithm, the proposed multiple asynchronous BO sensors tracking methodology is detailed in Section 4, followed by the implementation considerations of the proposed methods in Section 5. Section 6 demonstrates the experiment validation, followed by a conclusion in Section 7.

## 2. Problem Statement

This paper considers target tracking in challenging environments with imperfect target detection in a two dimensional (2D) surveillance space by using multiple asynchronous BO sensors. To focus on the main tracking challenges, the targets being tracked are assumed to be point targets, and the BO sensors used here are assumed to be with infinite sensor resolution, i.e., the resolutions of deployed BO sensors are small enough to distinguish from different objects in the angle measurement space, and each received angle measurement has only one source, either from target of interest or clutter. The necessary system models are mathematically formulated in this section.

### 2.1. Target Model

The target randomly appears and disappears in the surveillance space, consequently, its existence is a random event and modeled by a binary random variable. Denoting the target existence at time $t_{k}$ by $\chi_{k}$, which evolves as a first order Markov Chain in the time domain, and the probability that the target exists at time $t_{k}$ conditioned on it existed at time $t_{k-1}$ is mathematically described by [11]

(1) $$p_{11}=P\left(\chi_{k}|\chi_{k-1}\right)\approx 1-\frac{\Delta T_{k,k-1}}{T_{ave}},$$

where $\Delta T_{k,k-1}$ is the time interval of two consecutive scans, $T_{ave}$ denotes the average target existence duration and usually $T_{ave}>>T_{k,k-1}$. In this paper, that the possibility of target birth was treated by the random track initialization procedure, thus the probability that target exists at time $t_{k}$ given that it did not exist at time $t_{k-1}$ is assumed to be zero, i.e.,

(2) $$p_{12}=P\left(\chi_{k}|{\bar{\chi }}_{k-1}\right)=0.$$

Once the target exists in the surveillance area, one needs to estimate its kinematic state. For the sake of simplicity and clarity, the dynamic model of the target of interest is assumed to be linear and described by

(3) $$x_{k}=F_{k,k-1}x_{k-1}+w_{k},$$

where the target kinematic state consists of 2D position $x_{k}^{p}$ and velocity $x_{k}^{v}$, i.e., $x_{k}={\left[{\left(x_{k}^{p}\right)}^{T}\;{\left(x_{k}^{v}\right)}^{T}\right]}^{T}$, and $w_{k}$ is the process noise, which is modeled by the additive white Gaussian noise, with zero mean and covariance $Q_{k,k-1}$,

(4) $$Q_{k,k-1}=q\left[\begin{array}{cc} \frac{T_{k,k-1}^{3}}{3} & \frac{T_{k,k-1}^{2}}{2}\\ \frac{T_{k,k-1}^{2}}{2} & T_{k,k-1} \end{array}\right]⊗I_{2},$$

where *q* denotes the power spectral density, ⊗ is the Kronecker product, $I_{2}$ is the 2D identity matrix. $F_{k,k-1}$ denotes the dynamic state transition matrix from time $t_{k-1}$ to $t_{k}$, and given as

(5) $$F_{k,k-1}=\left[\begin{array}{cc} 1 & \Delta T_{k,k-1}\\ 0 & 1 \end{array}\right]⊗I_{2}.$$

The hybrid target state $\left(\chi_{k},x_{k}\right)$ modeled the above attempts to fully describe the statistics about the target behavior, wherein, the probability of target existence $\chi_{k}$ is used as an efficient track quality measure for track management, the kinematic state $x_{k}$ is only defined conditioning on the target existence $\chi_{k}$.

### 2.2. Sensor Model

At each time *k*, sensor *s* receives a random set of measurements $Z_{k}^{s}$ with set cardinal number $M_{k}^{s}$, denoting the *i*th measurement of $Z_{k}^{s}$ by $Z_{k,i}^{s}$. The measurement origin is unknown, because it may originate from the targets of interest or clutter. Denoting the set of received measurements up to and including time $t_{k}$ by $Z^{k,s}$. Both the target measurement model and clutter measurement model are defined below.

#### 2.2.1. Target Measurement

At time $t_{k}$, each sensor returns a single measurement $y_{k}$ for each target of interest with a detection probability $P_{D}$. A BO sensor can only measure the angle information of its line-of-sight in a 2D surveillance area, i.e., $y_{k}=\left[\theta_{k}\right]$, thus, the target measurement equation is a nonlinear function of the target and sensor kinematic state and given by

(6) $$y_{k}=h\left(x_{k},s_{k}\right)+v_{k}=\tan^{-1}\frac{y_{k}-y_{k}^{s}}{x_{k}-x_{k}^{s}}+v_{k},$$

where $s_{k}={\left[{\left(s_{k}^{p}\right)}^{T}\;{\left(s_{k}^{v}\right)}^{T}\right]}^{T}={\left[x_{k}^{s}\;y_{k}^{s}\;{\dot{x}}_{k}^{s}\;{\dot{y}}_{k}^{s}\right]}^{T}$ denotes the kinematic state of sensor *s* at time $t_{k}$, with ${\;}^{T}$ denoting the transpose, $v_{k}$ is the sensor noise usually described as additive white Gaussian distribution, with zero mean and known covariance $R_{k}$.

#### 2.2.2. Clutter Measurement

In practice, in addition to measurements originated from targets of interest, at time $t_{k}$, the sensor also returns a set of clutter measurements. The number of clutter measurements at each time $t_{k}$ is random and usually follows a Poisson distribution, the intensity of each clutter measurement $Z_{k,i}^{s}$ in the surveillance is termed as clutter measurement density and denoted by $\rho (Z_{k,i}^{s})$, which is usually assumed to be known but can also be estimated [30].

## 3. Overview of Automatic Target Tracking in Challenging Environments

The integrated probabilistic data association (IPDA) algorithm was validated and proved to be an effective approach for automatic target tracking in the presence of clutter disturbance and target misdetection. Since the proposed methodology composes of two parts: the local pseudo tracking and the central fusion, both of which need to deal with the problems of clutter measurement disturbance and target misdetection, the existing IPDA algorithm reviewed in this section is deployed as the cornerstone of the proposed algorithms introduced in Section 4. As a result, the content presented in this section is a prerequisite for introducing the algorithms developed in Section 4.

In the IPDA, the hybrid state $\left(\chi_{k-1},x_{k-1}\right)$ at time $t_{k-1}$ is mathematically described by a posterior probability density function (pdf) $p(\chi_{k-1},x_{k-1}|Z^{k-1})$, consisting of the probability of target existence $P(\chi_{k-1}|Z^{k-1})$ and the posterior pdf of the target kinematic state $p(x_{k-1}|\chi_{k-1},Z^{k-1})$ at time $t_{k-1}$, i.e.,

(7) $$p\left(\chi_{k-1},x_{k-1}|Z^{k-1}\right)=p\left(x_{k-1}|\chi_{k-1},Z^{k-1}\right)P\left(\chi_{k-1}|Z^{k-1}\right),$$

with

(8) $$p\left(x_{k-1}|\chi_{k-1},Z^{k-1}\right)\approx N\left(x_{k-1};{\hat{x}}_{k-1|k-1},P_{k-1|k-1}\right),$$

where $N(x_{k-1};{\hat{x}}_{k-1|k-1},P_{k-1|k-1})$ denotes the Gaussian distribution with mean ${\hat{x}}_{k-1|k-1}$ and its error covariance $P_{k-1|k-1}$.

The IPDA recursively updates the posterior pdf of hybrid state from time $t_{k-1}$ to time $t_{k}$ based on system models defined in Section 2 and sensor measurements received at time $t_{k}$. For simplicity, in the rest of the paper, the pdf of target kinematic state is implicitly conditioned on the target existence, i.e., $p\left(x_{k-1}|\chi_{k-1},Z^{k-1}\right)\equiv p\left(x_{k-1}|Z^{k-1}\right)$. One IPDA tracking cycle usually consists of track hybrid state prediction, gating and likelihood, data association, track hybrid state update, and is introduced in detail in the rest of this section.

### 3.1. Track Hybrid State Prediction

The predicted track hybrid state at time $t_{k-1}$ is denoted by $p(\chi_{k},x_{k}|Z^{k-1})$ and can be decomposed into two parts,

(9) $$p\left(\chi_{k},x_{k}|Z^{k-1}\right)=p\left(x_{k}|Z^{k-1}\right)P\left(\chi_{k}|Z^{k-1}\right),$$

where the predicted probability of target existence is obtained by

(10) $$P\left(\chi_{k}|Z^{k-1}\right)=p_{11}P\left(\chi_{k-1}|Z^{k-1}\right),$$

and the predicted target kinematic state pdf is

(11) $$p\left(x_{k}|Z^{k-1}\right)=N\left(x_{k};{\hat{x}}_{k|k-1},P_{k|k-1}\right),$$

where $N\left(x_{k};{\hat{x}}_{k|k-1},P_{k|k-1}\right)$ denotes the predicted Gaussian with mean ${\hat{x}}_{k|k-1}$ and corresponding covariance $P_{k|k-1}$, calculated by

(12) $${\hat{x}}_{k|k-1}=F_{k,k-1}{\hat{x}}_{k-1|k-1},$$

(13) $$P_{k|k-1}=F_{k,k-1}P_{k-1|k-1}F_{k,k-1}^{T}+Q_{k,k-1}.$$

### 3.2. Gating and Likelihood

To save computation and storage resources, the elliptical gating technique is used to select a set of measurements for track update.

(14) $${\left(Z_{k,i}-h\left({\hat{x}}_{k|k-1}\right)\right)}^{T}{\left(S_{k}\right)}^{-1}\left(Z_{k,i}-h\left({\hat{x}}_{k|k-1}\right)\right)⩽g,$$

with *g* is the gating threshold.

(15) $$S_{k}=H_{k}P_{k|k-1}H_{k}^{T}+R_{k},$$

where the measurement Jacobian matrix is given by

(16) $$H_{k}=\frac{\partial h\left(x_{k}\right)}{\partial x_{k}}/x_{k}={\hat{x}}_{k|k-1}.$$

After gating procedure, a subset $z_{k}$ of sensor received measurements $Z_{k}$ at time $t_{k}$ is validated, with $z_{k}={\left\{z_{k,i}\right\}}_{i=1}^{m_{k}}$. The likelihood of the selected measurement $z_{k,i}$ is thus calculated by

(17) $$p_{k,i}\equiv p\left(z_{k,i}|Z^{k-1}\right)=\frac{N\left(z_{k,i};h\left({\hat{x}}_{k|k-1}\right),S_{k}\right)}{P_{G}},$$

where $P_{G}$ denotes the gating probability, $N\left(z_{k,i};h\left({\hat{x}}_{k|k-1}\right),S_{k}\right)$ is the Gaussian distribution of $z_{k,i}$ with mean $h\left({\hat{x}}_{k|k-1}\right)$ and covariance $S_{k}$.

### 3.3. Data Association

Since the measurement origin is uncertain, one needs to evaluate all possibilities of validated measurement’ origins, the association probability that each measurement $z_{k,i}$ originates from target by

(18) $$\beta_{k,i}=\frac{1}{\delta_{k}}\left\{\begin{array}{cc} \frac{P_{D}P_{G}p_{k,i}}{\rho_{k,i}}, & i>0\\ 1-P_{D}P_{G}, & i=0, \end{array}\right.$$

where $P_{D}$ is the target detection probability, $\rho_{k,i}$ is the clutter measurement density of $z_{k,i}$. i=0 denotes that none of the validated measurements $z_{k}$ originated from the target, with the likelihood ratio defined by

(19) $$\delta_{k}=1-P_{D}P_{G}+P_{D}P_{G}\sum_{i=1}^{m_{k}}\frac{p_{k,i}}{\rho_{k,i}}.$$

### 3.4. Track Hybrid State Estimation

The updated track hybrid state is composed of two parts,

(20) $$p\left(\chi_{k},x_{k}|Z^{k}\right)=p\left(x_{k}|Z^{k}\right)P\left(\chi_{k}|Z^{k}\right),$$

where the updated probability of target existence is calculated by

(21) $$P\left(\chi_{k}|Z^{k}\right)=\frac{\delta_{k}P\left(\chi_{k}|Z^{k-1}\right)}{1-\left(1-\delta_{k}\right)P\left(\chi_{k}|Z^{k-1}\right)},$$

and the updated kinematic state pdf is still represented by a single Gaussian,

(22) $$p\left(x_{k}|Z^{k}\right)\approx N\left(x_{k};{\hat{x}}_{k|k},P_{k|k}\right),$$

where $N\left(x_{k};{\hat{x}}_{k|k},P_{k|k}\right)$ is the updated Gaussian of $x_{k}$ with mean ${\hat{x}}_{k|k}$ and covariance $P_{k|k}$, which is a Gaussian mixture of all the kinematic states updated using validated measurements $z_{k}$, i.e.,

(23) $$\left[{\hat{x}}_{k|k},P_{k|k}\right]=\mathrm{Gmix}{\left({\hat{x}}_{k|k,i},P_{k|k,i},\beta_{k,i}\right)}_{i=0}^{m_{k}},$$

with Gmix denotes the standard Gaussian mixture operation, $\beta_{k,i}$ is the data association probability obtained in Equation (18), ${\hat{x}}_{k|k,i}$ and $P_{k|k,i}$ are calculated as

(24) $$\left[{\hat{x}}_{k|k,i},P_{k|k,i}\right]=KF_{U}\left({\hat{x}}_{k|k-1},P_{k|k-1},z_{k,i},S_{k},H_{k}\right).$$

where $KF_{U}$ denotes the standard update procedure of the Kalman filter, the predicted mean ${\hat{x}}_{k|k-1}$ and covariance $P_{k|k-1}$ are given in Equations (12) and (13), the innovation covariance $S_{k}$ and measurement Jacobian matrix $H_{k}$ calculated in Equations (15) and (16). The recursively updated probability of target existence serves as a track quality measure used for track management, including timely recognizing true tracks then robustly maintaining them, and also quickly identifying false tracks not following any targets of interest thereafter deleting them from the memory.

## 4. Distributed Target Tracking in Challenging Environments Using Multiple Asynchronous BO Sensors

### 4.1. Framework of Proposed Methodology

**Figure 1:**
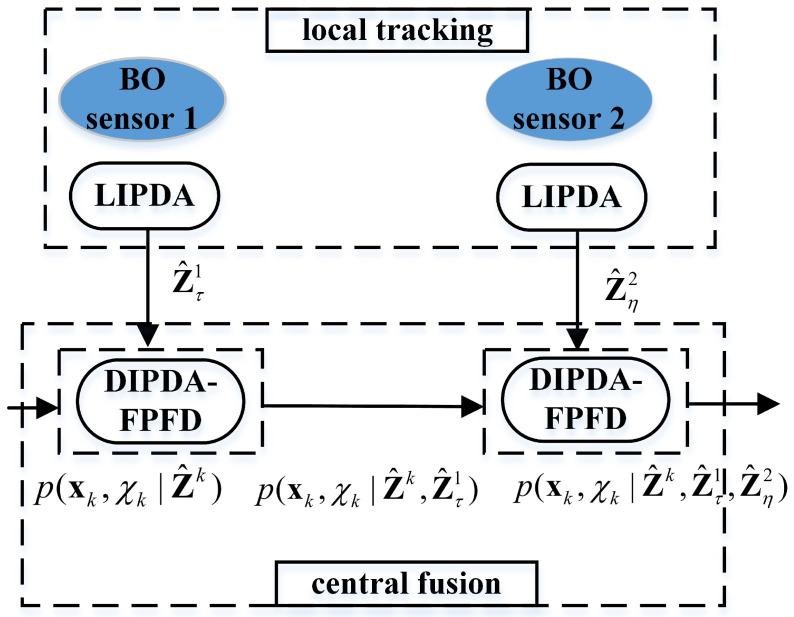
Framework of the proposed methodology.

A novel distributed fusion architecture for target tracking in challenging environments using multiple asynchronous BO sensors is proposed in this section. As can be seen from Figure 1, the framework of the proposed methodology consists of two steps: (1) firstly, a local IPDA (LIPDA) algorithm for local tracking in the presence of clutter measurement disturbance and target misdetection is used for each BO sensor. This is done to not only eliminate most of the clutter measurements which intensively reduces communication bandwidth between the local sensor and fusion center, but also tangibly refine target information; (2) secondly, the fusion center operates the proposed distributed IPDA-forward prediction fusion and decorrelation (DIPDA-FPFD) method for central fusion with OOS information aimed at obtaining maximum tracking performance improvement. Please note that the OOS information used for central fusion are actually sets of refined bearing measurements, which are transmitted by local BO sensors after implementing the LIPDA.

A more specific illustration on the proposed distributed tracking approach (taking two BO sensors as an example) is presented here. As shown in Figure 1, sensor 1 and 2 deploy the LIPDA for pseudo tracking using individually received raw measurements at time $t_{\tau }$ and $t_{\eta }$, respectively, and output two sets of refined bearing measurements ${\hat{Z}}_{\tau }^{1}$ and ${\hat{Z}}_{\eta }^{2}$, which arrive in the fusion center at current time $t_{\tau }$ and are regarded as OOS information due to $t_{\tau }<t_{\eta }<t_{k}$. The filtered central track hybrid state pdf $p(x_{k},\chi_{k}|{\hat{Z}}^{k})$ (obtained by filtering sets of refined bearing measurements gathered from all local BO sensors up to and including time $t_{k}$, i.e., ${\hat{Z}}^{k}$), is then sequentially updated via the proposed DIPDA-FPFD algorithm using the OOS information ${\hat{Z}}_{\tau }^{1}$ and ${\hat{Z}}_{\eta }^{2}$, respectively, resulting in an improved central track hybrid state estimation pdf $p(x_{k},\chi_{k}|{\hat{Z}}^{k},{\hat{Z}}_{\tau }^{1},{\hat{Z}}_{\eta }^{2})$.

### 4.2. Local IPDA (LIPDA)

Since a single BO sensor can only measure the bearing information of the line-of-sight (LOS) from the sensor to the target, the target’s kinematic state usually becomes unobservable (the case that sensor outmaneuvers the target to achieve the observability is not considered here), and the target measurement is nonlinear with respect to the target kinematic state. Instead of tracking the target kinematic state, the target measurement state is tracked in each local BO sensor using the proposed LIPDA algorithm, consequently, the measurement state can be completely (or partly) observed. The measurement tracking in the local sensors is called the pseudo tracking in this paper to differentiate from the conventional kinematic track tracking. Denoting the local pseudo track state (also termed as measurement state) at time $t_{k}$ by $B={\left[\theta_{k}\;{\dot{\theta }}_{k}\right]}^{T}$, where $\theta_{k}$ and ${\dot{\theta }}_{k}$ denote the bearing and bearing rate (the first derivative of bearing with respect to time), respectively. The measurement state propagation is modeled as

(25) $$B_{k}={\tilde{F}}_{k,k-1}B_{k-1}+{\tilde{w}}_{k},$$

where ${\tilde{F}}_{k,k-1}$ is the measurement state propagation matrix from time $t_{k-1}$ to $t_{k}$ and given by

(26) $${\tilde{F}}_{k,k-1}=\left[\begin{array}{cc} 1 & \Delta T_{k,k-1}\\ 0 & 1 \end{array}\right],$$

${\tilde{w}}_{k}$ is the measurement state process noise used to partially compensate the linearized propagation model error, and is approximated as the projection of the target kinematic state process noise described in Equation (3) [30],

(27) $${\tilde{w}}_{k}\approx \frac{\partial B_{k}\left(x_{k},s_{k}\right)}{\partial x_{k}}w_{k},$$

which is assumed to be a Gaussian with zero mean and covariance calculated by

(28) $${\tilde{Q}}_{k,k-1}\left(x_{k},s_{k}\right)=\frac{\partial B_{k}\left(x_{k},s_{k}\right)}{\partial x_{k}}Q_{k,k-1}{\left(\frac{\partial B_{k}\left(x_{k},s_{k}\right)}{\partial x_{k}}\right)}^{T}.$$

As can be seen above, ${\tilde{Q}}_{k,k-1}$ is a function of $x_{k}$ and $s_{k}$. In this BO sensor system, $x_{k}$ cannot be uniquely determined given $B_{k}$, $s_{k}$ and $Q_{k,k-1}$. Consequently, a reasonable way to obtain ${\tilde{Q}}_{k,k-1}$ is to find the biggest trace of ${\tilde{Q}}_{k,k-1}\left(x_{k},s_{k}\right)$ subject to the constraint that the local measurement state equals $B_{k}$ [30], i.e.,

(29) $${\tilde{Q}}_{k,k-1}=\max_{x_{k}:B_{k}\left(x_{k},\;s_{k}\right)=B_{k}}tr\left({\tilde{Q}}_{k,k-1}\left(x_{k},s_{k}\right)\right).$$

The Jacobian of local measurement state $B_{k}\left(x_{k},s_{k}\right)$ with respect to $x_{k}$ is

(30) $$\frac{\partial B_{k}\left(x_{k},s_{k}\right)}{\partial x_{k}}=\left[\begin{array}{cc} \frac{\partial \theta_{k}}{\partial x_{k}^{p}} & \frac{\partial \theta_{k}}{x_{k}^{v}}\\ \frac{\partial {\dot{\theta }}_{k}}{\partial x_{k}^{p}} & \frac{\partial {\dot{\theta }}_{k}}{\partial x_{k}^{v}} \end{array}\right].$$

Maximizing the trace of ${\tilde{Q}}_{k,k-1}\left(x_{k},s_{k}\right)$ is equivalent to maximize the sum of main diagonal elements of ${\tilde{Q}}_{k,k-1}\left(x_{k},s_{k}\right)$, subject to the local measurement state at time $t_{k}$ equals to $B_{k}$. As can be seen from Equation (28), the value of each main diagonal element in ${\tilde{Q}}_{k,k-1}\left(x_{k},s_{k}\right)$ is dominated by the main diagonal elements of $Q_{k,k-1}$, thus, maximizing the trace of ${\tilde{Q}}_{k,k-1}\left(x_{k},s_{k}\right)$ is equivalent to maximize the coefficient vectors of the main diagonal elements of $Q_{k,k-1}$, i.e., maximizing vectors $\frac{\partial \theta_{k}}{\partial x_{k}^{p}}$ and $\frac{\partial {\dot{\theta }}_{k}}{\partial x_{k}^{v}}$.

After mathematical transformation and simplification (see detailed derivation in the Appendix A), each elements of the Jacobian matrix above is calculated by

(31) $$\frac{\partial \theta_{k}}{\partial x_{k}^{p}}=\frac{i_{x}^{T}\cos\theta_{k}-i_{x}^{T}}{r_{\min}\left|\sin\theta_{k}\right|},$$

(32) $$\frac{\partial \theta_{k}}{x_{k}^{v}}=0_{1\times 2},$$

(33) $$\begin{array}{c} \frac{\partial {\dot{\theta }}_{k}}{\partial x_{k}^{p}}=\left\{\begin{array}{cc} \frac{2+3\cos\theta_{k}-\cos^{3}\theta_{k}}{r_{\min}^{2}\sin^{3}\theta }\left(v_{\max}^{t}i_{x}^{T}+v_{\max}^{s}i_{s}^{T}\right) & \theta_{k}\in \left(0,\pi \right)\\ \frac{3\cos^{3}\theta_{k}+4\cos^{2}\theta_{k}-3\cos\theta_{k}-2}{r_{\min}^{2}\sin^{3}\theta_{k}}\left(v_{\max}^{t}i_{x}^{T}+v_{\max}^{s}i_{s}^{T}\right) & \theta_{k}\in \left(\pi ,2\pi \right) \end{array}\right. \end{array},$$

(34) $$\frac{\partial {\dot{\theta }}_{k}}{\partial x_{k}^{v}}=\frac{i_{x}^{T}\cos\theta_{k}-i_{x}^{T}}{r_{\min}\left|\sin\theta_{k}\right|},$$

where $i_{x}$ is the unit vector of the X-axis of the sonar *s* local Cartesian coordinate ($X_{s}O_{s}Y_{s}$ defined in Section 6.2), $i_{s}$ is the unit vector of the sonar *s* position vector in the global Cartesian coordinate (XOY defined in Section 6.2), $0_{1\times 2}$ denotes the zero matrix with dimension 1×2, $r_{min}$ is the minimum sonar detection range, $v_{\max}^{t}$ and $v_{\max}^{s}$ denote the maximum velocity of target and sonar, respectively.

Since the local BO sensor can only measure the bearing measurement, the local measurement equation is a linear function of the local measurement state,

(35) $$\theta_{k}={\tilde{H}}_{k}B_{k}+v_{k},$$

where $v_{k}$ is the additive white Gaussian noise with zero mean and covariance $R_{k}$, and ${\tilde{H}}_{k}=\left[1\;0\right]$ is the measurement matrix.

In each local sensor, a pseudo track hybrid state $\left(\chi_{k},B_{k}\right)$ is recursively estimated by deploying the LIPDA algorithm, wherein, the main body of the LIPDA method is the same as the IPDA algorithm reviewed in Section 3, except that the local tracking models need to be specified by Equations (25) and (35). After completing the tracking loop, one is able to obtain the posterior pdf of pseudo track hybrid state as

(36) $$p\left(\chi_{k},B_{k}\right)=p\left(B_{k}|Z^{k}\right)P\left(\chi_{k}|Z^{k}\right),$$

with the local measurement state assumed to be a single Gaussian,

(37) $$p\left(B_{k}|Z^{k}\right)=N\left(B_{k};{\hat{B}}_{k|k},\Gamma_{k|k}\right),$$

where $N\left(B_{k};{\hat{B}}_{k|k},\Gamma_{k|k}\right)$ is the updated Gaussian of local measurement state $B_{k}$, with mean ${\hat{B}}_{k|k}$ and corresponding covariance $\Gamma_{k|k}$.

The recursively updated probability of target existence $P\left(\chi_{k}|Z^{k}\right)$ is used as a pseudo track quality measure to operate the local pseudo track management aimed at excluding most of the existing false pseudo tracks which do not follow any target of interest. The probability of target existence is also used to confirm and maintain all the true pseudo track following targets. For details about the track management procedure, please refer to Section 5. Only the confirmed pseudo tracks are communicated to the fusion center for central fusion. Here, instead of the full local measurement state, only the bearing estimation and its corresponded error covariance are transmitted so as to further release the communication burden. As a result, after local pseudo tracking, each sensor transfers a set of refined bearing measurements to the fusion center, i.e., ${\hat{Z}}_{k}^{s}={\left\{\theta_{k,i}^{s},\sigma_{k,i}^{s}\right\}}_{i}$, with $\theta_{k,i}^{s}$ and $\sigma_{k,i}^{s}$ denoting the *i*th refined bearing measurement and its error covariance of sensor *s*, respectively.

**Remark** **1.** *In the fusion center, central tracks are initialized and then updated using refined bearing measurements transmitted from multiple local BO sensors. When a central track is sequentially updated by refined bearing measurements transmitted from different local BO sensors, the correlation between this central track (defined in the Cartesian kinematic state space) and the refined bearing measurements (defined in the angle state space) lies in two aspects: (1) due to the scan to scan dependence of refined bearing measurements from the same local pseudo track, the central track is correlated with the refined bearing measurements transmitted from the same BO sensor that contributes to initialize this central track, this correlation is additionally exacerbated by the recursive update of this central track scan to scan; (2) as can be seen in Equation (27), the process noise of the local pseudo state at different BO sensors is derived from the common Cartesian kinematic state process noise. Refined bearing measurements across different BO sensors are correlated, consequently, the central track is correlated with the refined bearing measurements transmitted from other BO sensors.*

The exact correlation model is thus complex (interested readers please refer to [31]), and any solution which uses the exact correlation model to improve the tracking performance is likely to be very complicated and of limited value. To keep the simplicity of the proposed algorithms, this correlation is completely ignored in this paper.

### 4.3. Distributed IPDA-Forward Prediction Fusion and Decorrelation (DIPDA-FPFD) Technique

**Figure 2:**
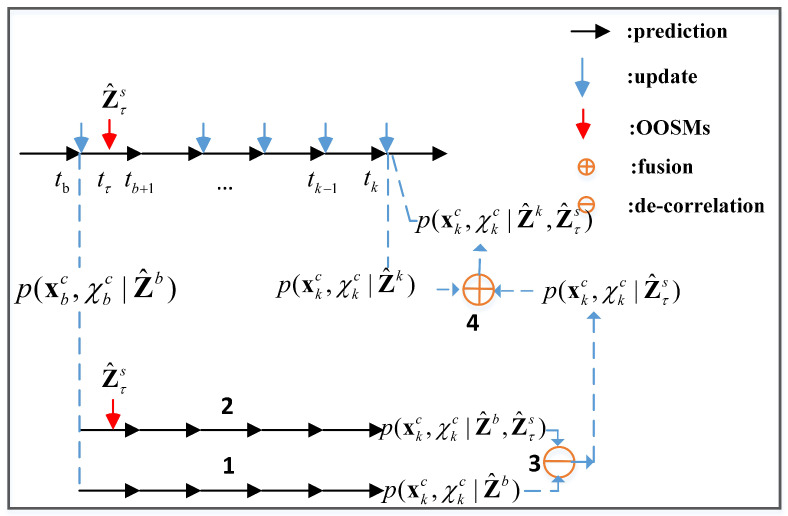
Flowchart of the proposed DIPDA-FPFD.

The main idea of the proposed DIPDA-FPFD methodology lies in the facts that it partially rules out the dependency in information between the central track hybrid state updated by OOS data and currently filtered central track hybrid state by employing a straightforward decorrelation procedure in the information space. The decorrelated track hybrid state estimation purely contributed by the OOS information is then fused with the filtered central track hybrid state to achieve an improved estimate. As demonstrated in Figure 2, the proposed DIPDA-FPFD algorithm composes of four steps: (1) forward predict the stored posterior hybrid state of central track *c* obtained at time $t_{b}$ to the current time $t_{k}$ to obtain $p(x_{k}^{c},\chi_{k}^{c}|{\hat{Z}}^{b})$; (2) forward predict the stored posterior hybrid state of central track *c* obtained at time $t_{b}$ to the OOS time $t_{\tau }$ ($t_{b}$ is one step prior to $t_{\tau }$) to obtain $p(x_{\tau }^{c},\chi_{\tau }^{c}|{\hat{Z}}^{b})$, which is then updated using OOS information ${\hat{Z}}_{\tau }^{s}$ transmitted by sensor *s* with time stamp $t_{\tau }$, and the obtained posterior hybrid state $p(x_{\tau }^{c},\chi_{\tau }^{c}|{\hat{Z}}^{b},{\hat{Z}}_{\tau }^{s})$ is forward predicted to current time $t_{k}$ to get the $p(x_{k}^{c},\chi_{k}^{c}|{\hat{Z}}^{b},{\hat{Z}}_{\tau }^{s})$; (3) decorrelate the obtained $p(x_{k}^{c},\chi_{k}^{c}|{\hat{Z}}^{b})$ and $p(x_{k}^{c},\chi_{k}^{c}|{\hat{Z}}^{b},{\hat{Z}}_{\tau }^{s})$ in the information space in order to eliminate the common information accumulated till current time $t_{k}$. This is done to get the information purely contributed by OOS information ${\hat{Z}}_{\tau }^{s}$, i.e., $p(x_{k}^{c},\chi_{k}^{c}|{\hat{Z}}_{\tau }^{s})$;4) fuse the current-time filtered hybrid state $p(x_{k}^{c},\chi_{k}^{c}|{\hat{Z}}^{k})$ and $p(x_{k}^{c},\chi_{k}^{c}|{\hat{Z}}_{\tau }^{s})$ in the information space to obtain the eventual posterior hybrid state pdf $p(x_{k}^{c},\chi_{k}^{c}|{\hat{Z}}^{k},{\hat{Z}}_{\tau }^{s})$.

The aim of the proposed DIPDA-FPFD algorithm is to update the current-time filtered central track hybrid state pdf $p(x_{k}^{c},\chi_{k}^{c}|{\hat{Z}}^{k})$ using the OOS information ${\hat{Z}}_{\tau }^{s}$ so as to achieve an improved posterior hybrid state estimation $p(x_{k}^{c},\chi_{k}^{c}|{\hat{Z}}^{k},{\hat{Z}}_{\tau }^{s})$. Each of the four parts of the proposed DIPDA-FPFD is presented in detail in the rest of this section.

#### 4.3.1. Forward Predict Hybrid State without OOS Information from Time $t_{b}$ to $t_{k}$

The forward predicted hybrid state pdf without OOS information is mathematically described by

(38) $$p\left(x_{k}^{c},\chi_{k}^{c}|{\hat{Z}}^{b}\right)=P\left(\chi_{k}^{c}|{\hat{Z}}^{b}\right)p\left(x_{k}^{c}|{\hat{Z}}^{b}\right),$$

where $P(\chi_{k}^{c}|{\hat{Z}}^{b})$ denotes the forward predicted probability of target existence and is calculated by

(39) $$P\left(\chi_{k}^{c}|{\hat{Z}}^{b}\right)={\left(p_{11}\right)}^{\mathrm{int}\left(\frac{t_{k}-t_{b}}{\Delta T_{k,k-1}}\right)}P\left(\chi_{b}^{c}|{\hat{Z}}^{b}\right),$$

with int(a) denotes the integer part of *a*, $P(\chi_{b}^{c}|Z^{b})$ denotes the posterior probability of target existence at time $t_{b}$. The forward predicted kinematic state pdf $p(x_{k}^{c}|{\hat{Z}}^{b})$ is represented by a Gaussian, i.e.,

(40) $$p\left(x_{k}^{c}|{\hat{Z}}^{b}\right)=N\left(x_{k}^{c};{\bar{x}}_{k|b}^{c}.{\bar{P}}_{k|b}^{c}\right),$$

where its mean and estimated error covariance are obtained by

(41) $${\bar{x}}_{k|b}^{c}=F_{k,b}{\hat{x}}_{b|b}^{c},$$

(42) $${\bar{P}}_{k|b}^{c}=F_{k,b}P_{b|b}^{c}{\left(F_{k,b}\right)}^{T}+Q_{k,b},$$

where ${\hat{x}}_{b|b}^{c}$ and $P_{b|b}^{c}$ are the mean and its estimation covariance of posterior kinematic state pdf of central track at time $t_{b}$, state propagation matrix $F_{k,b}$ and process noise $Q_{k,b}$ are defined in Equations (5) and (4), respectively.

#### 4.3.2. Forward Predict Hybrid State with OOS Information from Time $t_{\tau }$ to $t_{k}$

Firstly predict the previously stored central track hybrid state $p(x_{b}^{c},\chi_{b}^{c}|{\hat{Z}}^{b})$ at time $t_{b}$ to $t_{\tau }$ so as to obtain the predicted hybrid state $p(x_{\tau }^{c},\chi_{\tau }^{c}|{\hat{Z}}^{b})$. It is then followed by implementing the IPDA-EKF algorithm (reviewed in Section 3) to obtained the updated posterior hybrid state pdf using the OOS information set ${\hat{Z}}_{\tau }^{s}$ at time $t_{\tau }$, resulted in $p(x_{\tau }^{c},\chi_{\tau }^{c}|{\hat{Z}}^{b},{\hat{Z}}_{\tau }^{s})$, which consists of

(43) $$p\left(\chi_{\tau }^{c},x_{\tau }^{c}|{\hat{Z}}^{b},{\hat{Z}}_{\tau }^{s}\right)=P\left(\chi_{\tau }^{c}|{\hat{Z}}^{b},{\hat{Z}}_{\tau }^{s}\right)p\left(x_{\tau }^{c}|{\hat{Z}}^{b},{\hat{Z}}_{\tau }^{s}\right),$$

with the posterior kinematic state pdf represented by a single Gaussian,

(44) $$p\left(x_{\tau }^{c}|{\hat{Z}}^{b},{\hat{Z}}_{\tau }^{s}\right)=N\left(x_{\tau }^{c};{\hat{x}}_{\tau |b,\tau }^{c},P_{\tau |b,\tau }^{c}\right),$$

The forward predicted central track hybrid state pdf is

(45) $$p\left(\chi_{k}^{c},x_{k}^{c}|{\hat{Z}}^{b},{\hat{Z}}_{\tau }^{s}\right)=P\left(\chi_{k}^{c}|{\hat{Z}}^{b},{\hat{Z}}_{\tau }^{s}\right)p\left(x_{k}^{c}|{\hat{Z}}^{b},{\hat{Z}}_{\tau }^{s}\right),$$

with the probability of target existence at time $t_{\tau }$ predicted by

(46) $$P\left(\chi_{k}^{c}|{\hat{Z}}^{b},{\hat{Z}}_{\tau }^{s}\right)={\left(p_{11}\right)}^{\mathrm{int}\left(\frac{t_{k}-t_{\tau }}{\Delta T_{k,k-1}}\right)+1}P\left(\chi_{\tau }^{c}|{\hat{Z}}^{b},{\hat{Z}}_{\tau }^{s}\right),$$

and the predicted kinematic state pdf at time $t_{k}$ represented by a single Gaussian

(47) $$p\left(x_{k}^{c}|\chi_{k}^{c},{\hat{Z}}^{b},{\hat{Z}}_{\tau }^{s}\right)=N\left(x_{k}^{c};{\bar{x}}_{k|b,\tau }^{c},{\bar{P}}_{k|b,\tau }^{c}\right),$$

with its mean and error covariance given by

(48) $${\bar{x}}_{k|b,\tau }^{c}=F_{k,\tau }{\hat{x}}_{\tau |b,\tau }^{c},$$

(49) $${\bar{P}}_{k|b,\tau }^{c}=F_{k,\tau }P_{\tau |b,\tau }^{c}{\left(F_{k,\tau }\right)}^{T}+Q_{k,\tau }.$$

#### 4.3.3. Decorrelate OOS Information Updated Hybrid State

To rule out the common information between the current-time filtered hybrid state and the OOS information updated hybrid state, $p(x_{k}^{c},\chi_{k}^{c}|{\hat{Z}}^{b},{\hat{Z}}_{\tau }^{s})$ is de-correlated by directly subtracting the duplicated information contained in $p(x_{k}^{c},\chi_{k}^{c}|{\hat{Z}}^{b})$, and one obtains the central track hybrid state solely updated by OOS information ${\hat{Z}}_{\tau }^{s}$ at time $t_{k}$, i.e.,

(50) $$p\left(\chi_{k}^{c},x_{k}^{c}|{\hat{Z}}_{\tau }^{s}\right)=P\left(\chi_{k}^{c}|{\hat{Z}}_{\tau }^{s}\right)p\left(x_{k}^{c}|{\hat{Z}}_{\tau }^{s}\right),$$

where

(51) $$P\left(\chi_{k}^{c}|{\hat{Z}}_{\tau }^{s}\right)=P\left(\chi_{k}^{c}|{\hat{Z}}^{b},{\hat{Z}}_{\tau }^{s}\right)-P\left(\chi_{k}^{c}|{\hat{Z}}^{b}\right),$$

its kinematic state pdf is represented by a single Gaussian, i.e., $p\left(x_{k}^{c}|\chi_{k}^{c},{\hat{Z}}_{\tau }^{s}\right)=N\left(x_{k}^{c};{\hat{x}}_{k|\tau }^{c},P_{k|\tau }^{c}\right)$, which is calculated in the information space,

(52) $${\left(P_{k|\tau }^{c}\right)}^{-1}={\left({\bar{P}}_{k|b,\tau }^{c}\right)}^{-1}-{\left({\bar{P}}_{k|b}^{c}\right)}^{-1},$$

(53) $${\left(P_{k|\tau }^{c}\right)}^{-1}{\hat{x}}_{k|\tau }^{c}={\left({\bar{P}}_{k|b,\tau }^{c}\right)}^{-1}{\bar{x}}_{k|b,\tau }^{c}-{\left({\bar{P}}_{k|b}^{c}\right)}^{-1}{\bar{x}}_{k|b}^{c}.$$

#### 4.3.4. Fuse the Current-Time Filtered Hybrid State Using OOS Information

The eventually fused central track hybrid state pdf contains the updated probability of target existence and the fused kinematic states pdf, i.e.,

(54) $$p\left(\chi_{k}^{c},x_{k}^{c}|{\hat{Z}}^{k},{\hat{Z}}_{\tau }^{s}\right)=P\left(\chi_{k}^{c}|{\hat{Z}}^{k},{\hat{Z}}_{\tau }^{s}\right)p\left(x_{k}^{c}|{\hat{Z}}^{k},{\hat{Z}}_{\tau }^{s}\right),$$

where the fused probability of target existence is calculated by

(55) $$P\left(\chi_{k}^{c}|{\hat{Z}}^{k},{\hat{Z}}_{\tau }^{s}\right)=P\left(\chi_{k}^{c}|{\hat{Z}}^{k}\right)+P\left(\chi_{k}^{c}|{\hat{Z}}_{\tau }^{s}\right)-P\left(\chi_{k}^{c}|{\hat{Z}}^{k}\right)P\left(\chi_{k}^{c}|{\hat{Z}}_{\tau }^{s}\right),$$

and its fused kinematic state pdf is still represented by a single Gaussian $p\left(x_{k}^{c}|{\hat{Z}}^{k},{\hat{Z}}_{\tau }^{s}\right)=N\left(x_{k}^{c};{\hat{x}}_{k|k,\tau }^{c},P_{k|k,\tau }^{c}\right)$, which is obtained by fusion in the information space,

(56) $${\left(P_{k|k,\tau }^{c}\right)}^{-1}={\left({\hat{P}}_{k|k}^{c}\right)}^{-1}+{\left(P_{k|\tau }^{c}\right)}^{-1},$$

(57) $${\left(P_{k|k,\tau }^{c}\right)}^{-1}{\hat{x}}_{k|k,\tau }^{c}={\left({\hat{P}}_{k|k}^{c}\right)}^{-1}{\hat{x}}_{k|k}^{c}+{\left(P_{k|\tau }^{c}\right)}^{-1}{\hat{x}}_{k|\tau }^{c}.$$

If there are OOS information from other sensors arrived at time $t_{k}$, the procedures in this subsection are repeated one sensor by one sensor. After updated with all the OOS information at time $t_{k}$, the fused hybrid states are renominated as $p\left(\chi_{k}^{c},x_{k}^{c}|{\hat{Z}}^{k},{\hat{Z}}_{\tau }^{s}\right)$ for next central track hybrid state fusion.

## 5. Implementation

### 5.1. Pseudo Track/Track Management

As far as track management is concerned, there is no difference in management for pseudo tracks in local sensor and tracks at the fusion center. Therefore, pseudo tracks and tracks will be termed as tracks in this subsection. Both true tracks following the targets of interest and false tracks not following any targets of interest are initiated and they survive in the subsequent recursions. As a consequence, an efficient track management technique is critically important, which is able to quickly confirm true tracks and maintain them in the sequel, and also to recognize false tracks as many as possible and then delete them from the memory. We use the recursively updated probability of target existence as a track quality measure to implement the real time track management.

Each initialized track is given a tentative status, once its recursively calculated probability of target existence exceeds a predefined confirmation threshold $\tau_{c}$, this track is upgraded to a confirmed status which indicates it is following the target of interest and thus maintained to be confirmed. A confirmed true track may become false alarm and terminated if its probability of target existence falls below a predefined termination threshold $\tau_{t}$, this may happen if the confirmed track is misled into following any clutter or target of non-interest. Additionally, a tentative track may also directly become a false track in a few scans after initialization. Once a track is declaimed to be a false track, it is deleted from memory.

### 5.2. Storage Consideration

When fusing the current-time filtered hybrid state using OOS information, the proposed DIPDA-FPFD algorithm needs to store the previously filtered hybrid state estimation of central tracks. The stored necessity information includes:

- $t_{i}$ where i=b,b+1,…,k−1,k, which requires k−b+1 scalars indicating the time stamp for which the central track is updated, with OOS information arrives at the fusion center between $t_{b}$ and $t_{b+1}$.
- ${\left({\hat{x}}_{i|i}^{c}\right)}_{i=b}^{i=k}$, requires m(k−b+1) scalars that indicates the mean of the filtered kinematic state of central track *c* from time $t_{k-b}$ to $t_{k}$, with *m* denotes the dimension of the kinematic state.
- ${\left({\hat{P}}_{i|i}^{c}\right)}_{i=b}^{i=k}$, requires (k−b+1)m(m+1)/2 scalars that indicates the error covariance of the filtered kinematic state estimation of central track *c* from time $t_{k-b}$ to $t_{k}$.

It is obvious that the tracking system requires to store tremendous information as the OOS lag increases. While in the real application the storage memory is usually limited, thus the maximum OOS lag ${\left(k-b\right)}_{\max}$ is predefined to enable to store previously filtered central track hybrid state information in a sliding time window.

## 6. Simulation Validation

**Figure 3:**
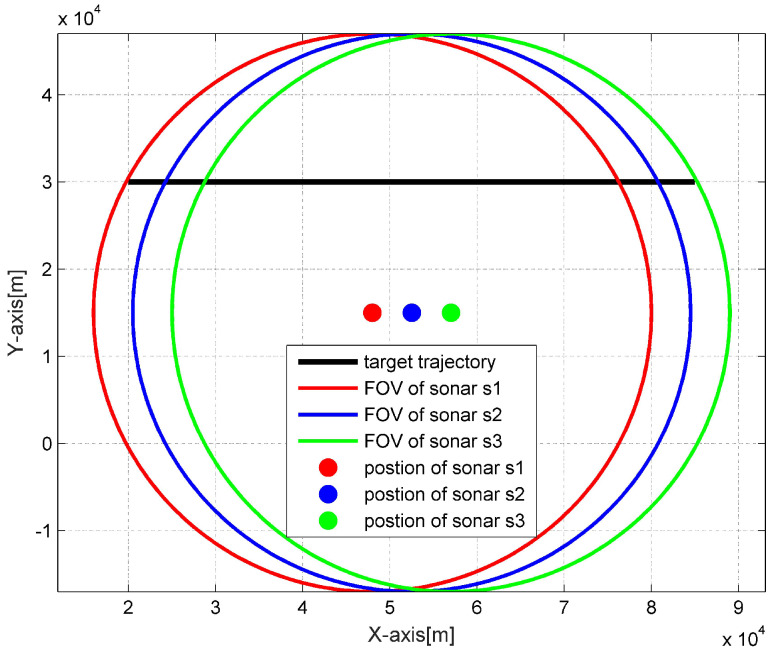
The target trajectory and surveillance areas of BO sonars.

We consider a 2D maritime target tracking scenario in challenging environments using three asynchronous BO sensors. As depicted in Figure 3, the target starts at position (20, 30) km and moves from the west to east with a nearly constant velocity 26 m/s, Three sonars are statically deployed at the positions (48, 15) km, (52.5, 15) km, (57, 15) km, with each surveillance range $r_{\max}=32\;\mathrm{km}$. The sonar received BO measurement is corrupted by a Gaussian noise with zero mean and variance $R={\left(2^{o}\right)}^{2}$, and the number of clutter measurements returned by each sonar at each scan follows a Poisson distribution with mean ${\bar{m}}_{c}$ and these clutter measurements are uniformly distributed between the bearing range of 0 to 2π, thus the clutter measurement density satisfies $\rho ={\bar{m}}_{c}/\left(2\pi \right)$. The detection probability $P_{D}$ of each sensor is assumed to be equal. The sampling interval of each sonar is same and equals to T=1 s, among the three sonars, the third sonar $s_{3}$ is designed to be asynchronous compared to the other two sonars, and transmits its local information to the fusion center with a random time delay *l*. As can be seen from Figure 3, the target firstly appears at the surveillance area of sonar $s_{1}$, then enters the surveillance area of sonar $s_{2}$ and $s_{3}$ successively, after collectively observed by three sonars for some time, the target moves out of the surveillance area of sonar $s_{1}$ at first, then out of sonar $s_{2}$ surveillance area, and finally disappears at the surveillance area of sonar $s_{3}$. The experiment repeats for 100 Monte Carlo runs, with 2500 s for each run duration.

To fully investigate the proposed methods, two experiments in the cases of different mean number of clutter measurements ${\bar{m}}_{c}$, target detection probability $P_{D}$, and OOS lag *l* are conducted in this section, i.e., (1) **case 1**: ${\bar{m}}_{c}=10$, $P_{D}=0.8$, l=1,3,10; (2) **case 2**: ${\bar{m}}_{c}=60$, $P_{D}=0.6$, l=1,3,10; (3) **case 3**: ${\bar{m}}_{c}=60$, $P_{D}=0.4$, l=1,3,10. Compared to case 1, cases 2 and 3 are more challenging with lower target detection probability and higher clutter measurement density, among the three cases, case 3 simulates the lowest target detection probability.

### 6.1. Local Tracking Results

In the local tracking, the measurement state is tracked so as to eliminate most of the clutter disturbances and also to improve the accuracy of the target information. Consequently, the numerical statistics results of local pseudo tracking are demonstrated below in tables. Please note that only the local tracking results of sensor $s_{2}$ are explicitly shown here, the results of other two sensors are quite similar to that of sensor $s_{2}$ and thus omitted.

As shown in Table 1, Table 2 and Table 3, the local tracking in sonar $s_{2}$ at case 1, 2, and 3 both initiates hundreds of thousands of tentative pseudo tracks whose total number over the entire experiment time increases exponentially, while, by using the efficient pseudo track management technique embedded in the proposed LIPDA, the total number of pseudo tracks eventually transmitted to the fusion center was reduced by 70%∼80% in case 1, by 30%∼80% in case 2, and by 12%∼40% in case 3, which greatly release the communication burden from the local sonar to fusion center, and also decrease the computation complexity in the fusion center.

**Figure 4:**
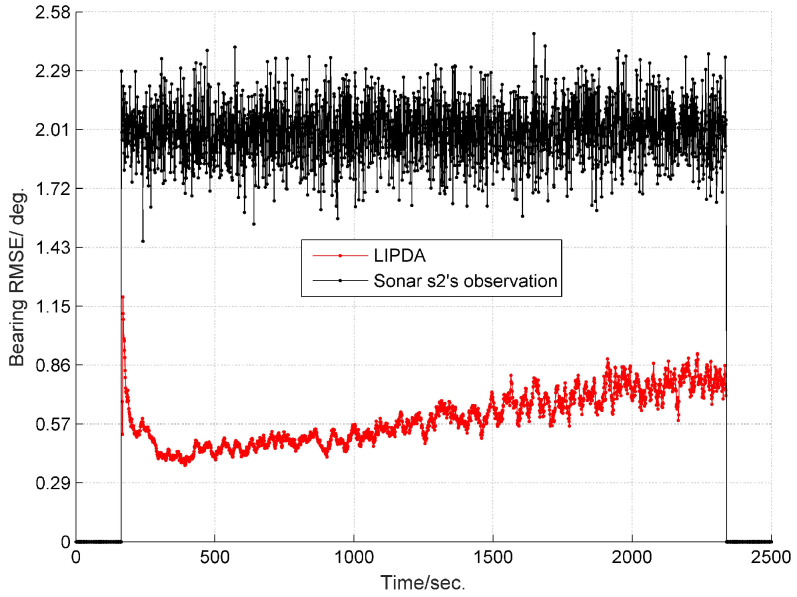
RMSE of estimated target bearing and the raw bearing observed by sonar $s_{2}$ at case 1.

**Figure 5:**
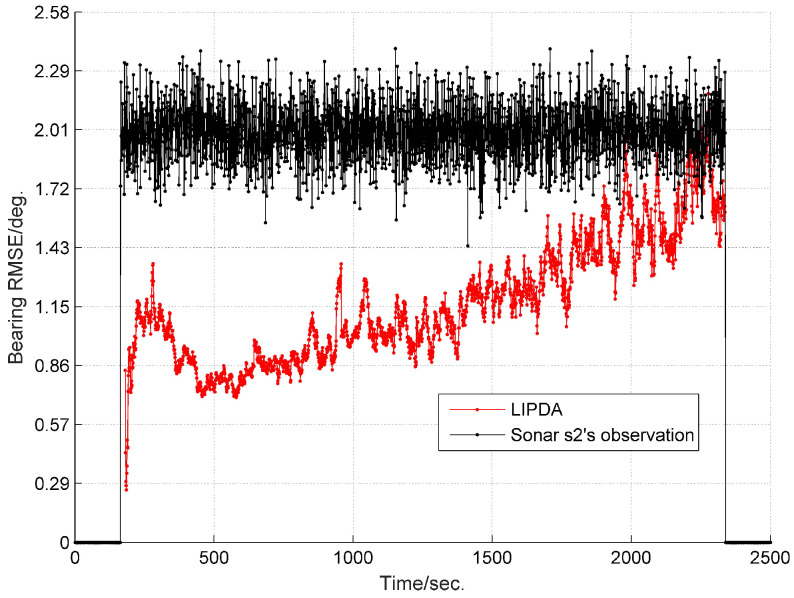
RMSE of estimated target bearing and the raw bearing observed by sonar $s_{2}$ at case 2.

**Figure 6:**
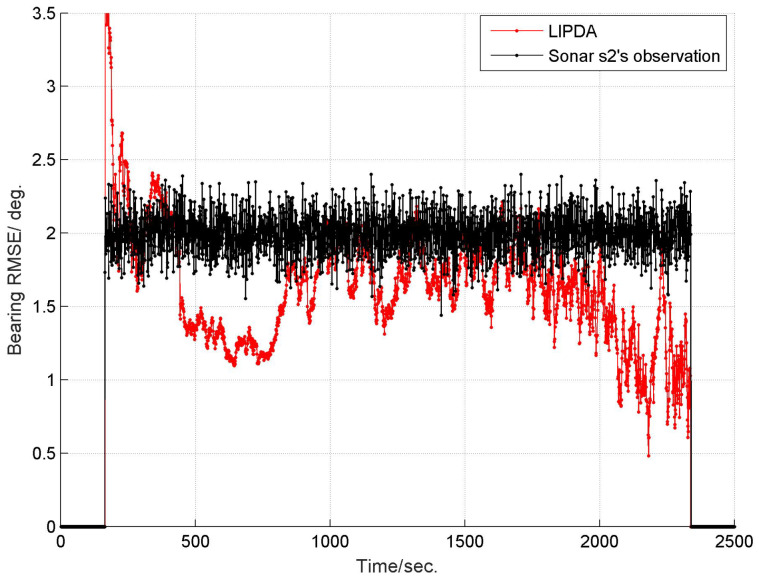
RMSE of estimated target bearing and the raw bearing observed by sonar $s_{2}$ at case 3.

The root mean square errors (RMSEs) of the estimated target bearing by the local tracking and directly observed by the sonar $s_{2}$ in cases 1, 2, and 3 are demonstrated in Figure 4, Figure 5 and Figure 6, respectively. As can be clearly seen, after operating the local pseudo tracking with proposed LIPDA, the accuracy of the target bearing measurement was substantially improved, i.e., the bearing bias is reduced by around $1.4^{{}^{\circ}}$ in case 1, by about $0.3^{{}^{\circ}}$∼$1.1^{{}^{\circ}}$ in case 2, and by near $0.1^{{}^{\circ}}$∼$1.0^{{}^{\circ}}$ in case 3. As can be seen from Figure 6, the proposed LIPDA method gives oscillating target bearing estimates in case 3, and shows trivial improvement over the raw observation at some instances. This is because the target detection probability in case 3 is too low for the proposed local tracking algorithm to always guarantee consistent and substantial pseudo tracking benefits.

### 6.2. Central Tracking Results

In the fusion center, the refined bearing measurements transmitted by each sonar are sequentially fused using the proposed DIPDA-FPFD algorithm, among them, the information transferred by sonar $s_{3}$ arrives in the fusion center with out of sequence. For fair comparison, the reprocessing method in [14] is enhanced by incorporating the distributed IPDA algorithm (termed as DIPDA-Re) for dealing with the problem of target misdetection and clutter disturbance. The DIPDA-Re serves as the upper performance benchmark since it delivers the best fusion performance via reprocessing the OOSMs in a chronological order. Please note that the DIPDA-Re method is not a realistic online multisensor OOSMs fusion algorithm because it needs to store all local pseudo tracks information from last fusion time till to next OOSMs coming, the fusion operation can be tremendously delayed and it also requires huge storage memory. Besides, the straightforward discarded approach proposed in [13] is also enhanced by enabling its track management capability in the presence of clutter and target misdetection and termed as DIPDA-D, which is implemented as the low benchmark.

**Figure 7:**
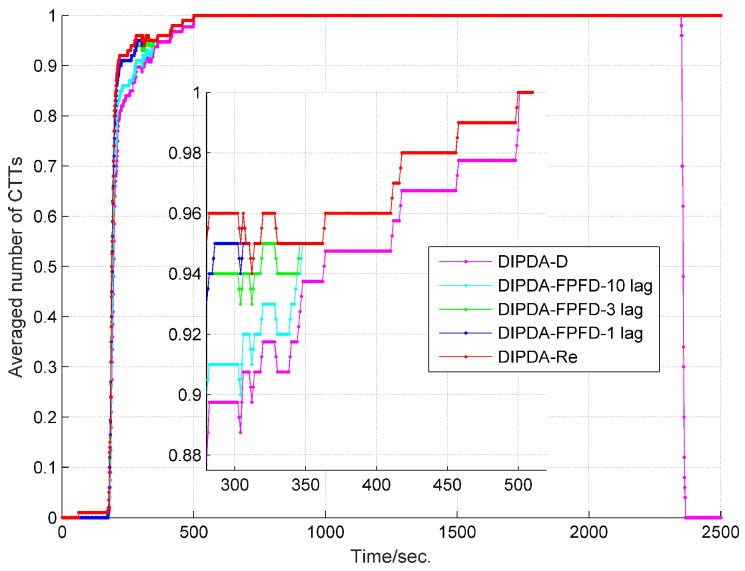
Averaged number of CTTs of DIPDA-D, DIPDA-Re, DIPDA-FPFD with different lags (case 1).

To fairly evaluate the track management performance among the three central fusion methods, the parameters of each method (including initial probability of target existence, confirmation threshold and termination threshold) are tuned to deliver similar number of confirmed false tracks (CFTs), then their averaged numbers of confirmed true tracks (CTTs) are compared. The averaged number of CTTs of each central fusion method in case 1 is shown in Figure 7, with each method delivering 1 CFT during the entire experiment time. At the beginning, it takes hundreds of seconds for each method to fully initiate all CTTs, the upper benchmark-DIPDA-Re gives the most averaged number of CTTs, followed by our proposed DIPDA-FPFD method with 1, 3, 10 lag, the averaged number of CTTs decreases as the OOS lag increases, indicating that our proposed method is a suboptimal solution, the low benchmark gives the least number of CTTs. At the end of the experiment, the DIPDA-D loses the target rapidly while the other methods still robustly maintain all the CTTs. The reason is that the target is out of the field of views (FOVs) of sonar $s_{1}$ and $s_{2}$, and only observed by the asynchronous sonar $s_{3}$, the DIPDA-D directly discards all the OOS information transmitted by sonar $s_{3}$, the PTEs of central tracks cannot be updated using new information thus rapidly reduce below the track termination threshold.

**Figure 8:**
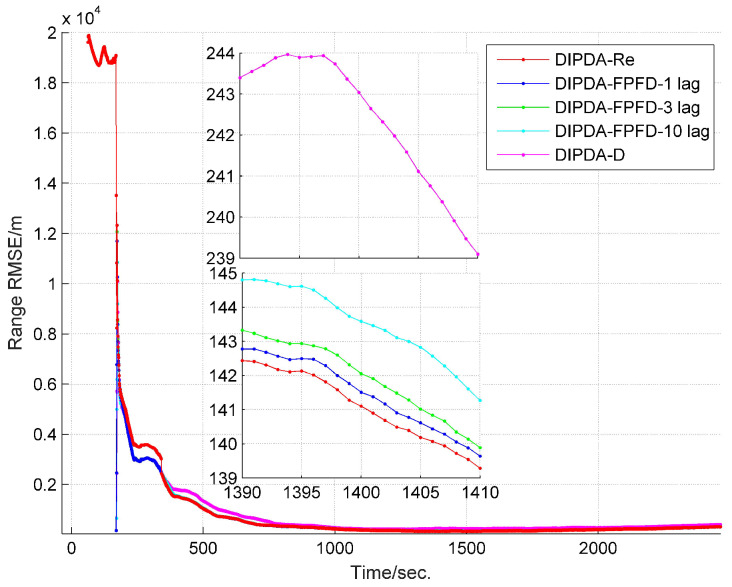
RMSE of estimated target range of DIPDA-D, DIPDA-Re, DIPDA-FPFD with different lags (case 1).

**Figure 9:**
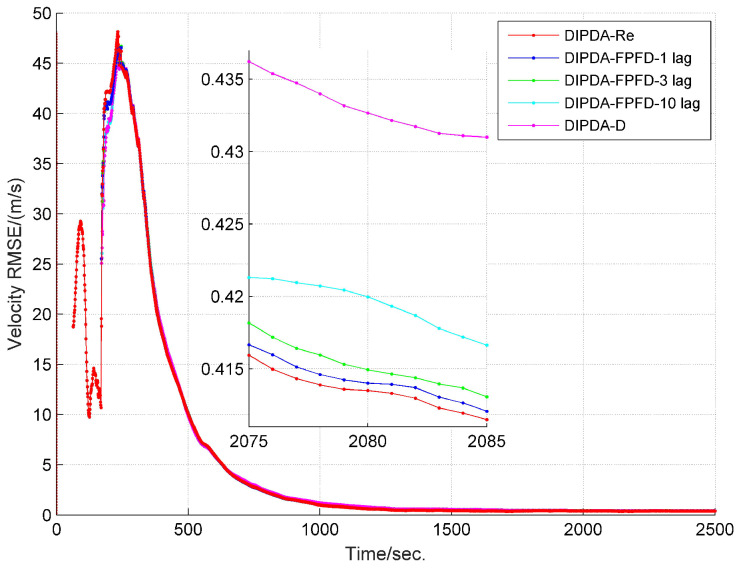
RMSE of estimated target velocity of DIPDA-D, DIPDA-Re, DIPDA-FPFD with different lags (case 1).

The range and velocity estimation RMSEs in case 1 are presented in Figure 8 and Figure 9. As can be seen, both the range and velocity estimation errors drastically reduce once the target enters the common FOV of sonar $s_{1}$ and $s_{2}$ which make the target kinematic state observable, and then converge to steady values. Among the three fusion methods, the DIPDA-D method delivers the biggest position and velocity estimation errors, because it directly discards valuable target information, the DIPDA-Re method gives the best tracking accuracy in both position and velocity estimation due to its reprocessing implementation on the OOS information, followed by our proposed DIPDA-FPFD method with 1, 3, 10 lag, the 10 lag case delivers slightly increased position and velocity estimation errors because of intensively increased OOS lag, but its performance is still comparable to the upper benchmark method.

**Figure 10:**
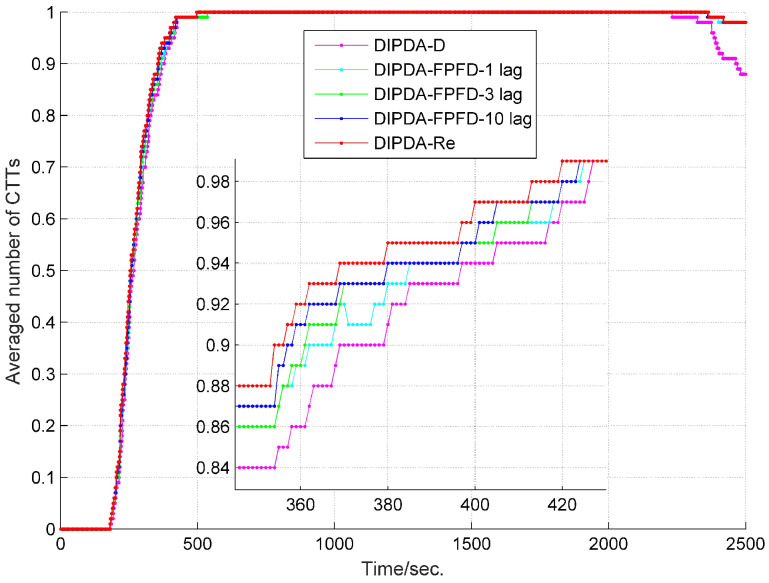
Averaged number of CTTs of DIPDA-D, DIPDA-Re, DIPDA-FPFD with different lags (case 2).

**Figure 11:**
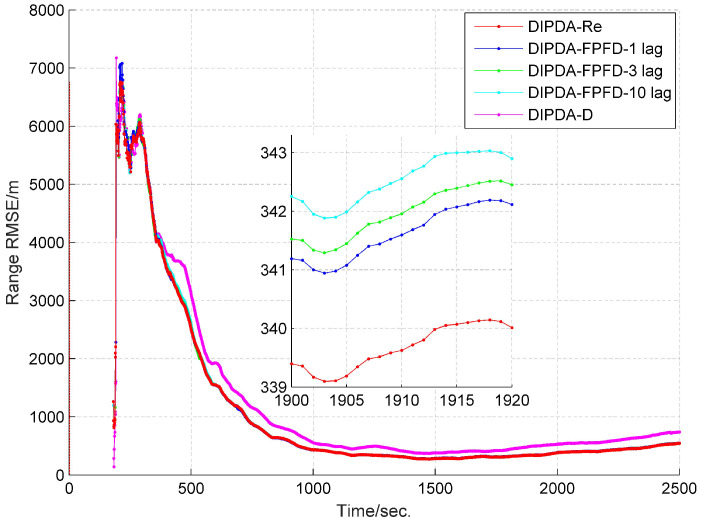
RMSE of estimated target range of DIPDA-D, DIPDA-Re, DIPDA-FPFD with different lags (case 2).

**Figure 12:**
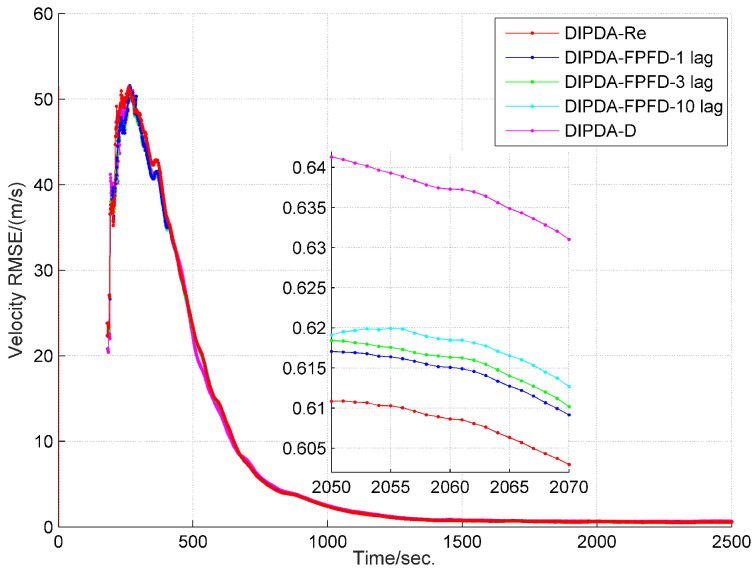
RMSE of estimated target velocity of DIPDA-D, DIPDA-Re, DIPDA-FPFD with different lags (case 2).

**Figure 13:**
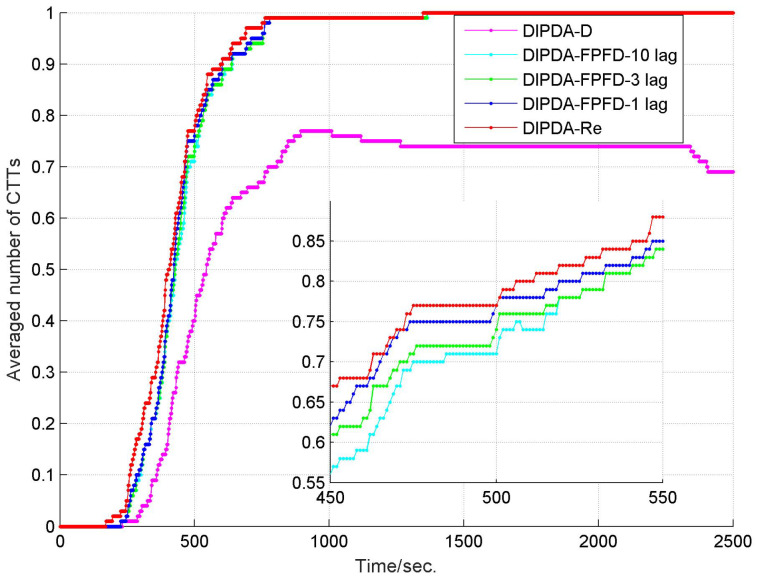
Averaged number of CTTs of DIPDA-D, DIPDA-Re, DIPDA-FPFD with different lags (case 3).

**Figure 14:**
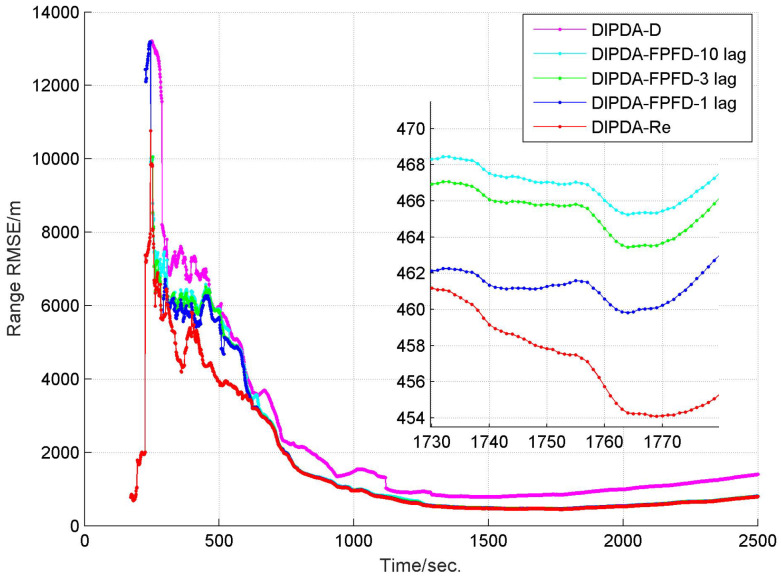
RMSE of estimated target range of DIPDA-D, DIPDA-Re, DIPDA-FPFD with different lags (case 3).

**Figure 15:**
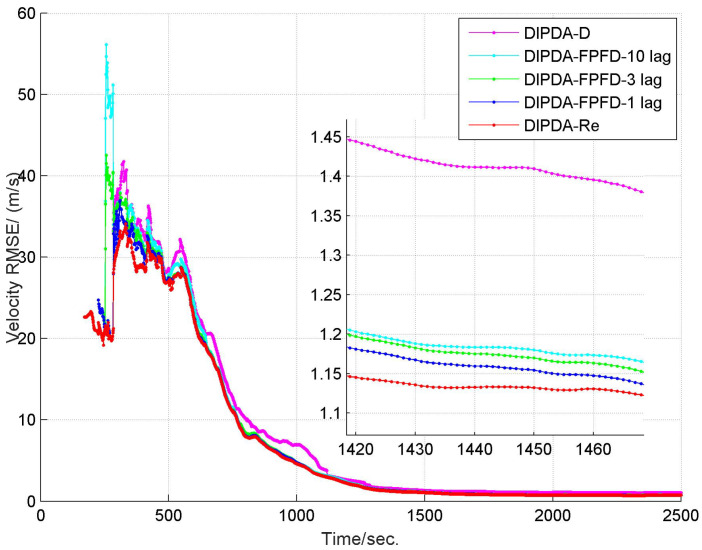
RMSE of estimated target velocity of DIPDA-D, DIPDA-Re, DIPDA-FPFD with different lags (case 3).

To verify the robustness of the proposed methods, another two much more challenging experiments are conducted. To give fair comparisons, the track management parameters of each method are tuned to deliver 3 and 7 CFTs for case 2 and case 3, respectively. As shown in Figure 10, it takes longer time for each central fusion method in cases 2 to fully initiate averaged number of CTTs compared to that in case 1, this is because case 2 is set to be with lower target detection probability and much worse clutter disturbance. The estimated target range and velocity RMSEs of case 2 are shown in Figure 11 and Figure 12, respectively. Besides, Figure 13 demonstrates the averaged number of CTTs of each compared method in case 3, which shows slower CTT initiating speed and fewer CTTs than that in case 2, due to further reduced target detection probability. The estimated target range and velocity RMSEs of case 3 are present in Figure 14 and Figure 15, respectively. Both cases 2 and 3 show deteriorated tracking accuracy compared to that of case 1 due to reduced target detection probability and increased clutter measurement disturbance. However, in terms of both track management and tracking accuracy, our proposed DIPDA-FPFD method with 1, 3, 10 lag still delivers comparable tracking results as the optimal benchmark DIPDA-Re approach, and intensively outperforms the DIPDA-D method.

Aside from tracking performance, the computational complexity and storage requirement of each method in case 2 are also compared in Table 4 and Table 5. All algorithms are implemented in MATLAB 2017b on system with Intel(R) Core(TM) i7-4700MQ, 2.40 GHz processor, 8GB memory and Windows 10 platform. The computational complexity of each algorithm is evaluated by its averaged elapsed time per scan. The storage requirement of each algorithm at every scan is evaluated using the number of scalars necessarily stored in the fusion center for tracking purpose. As shown in Table 4, the averaged elapsed time of the proposed DIPDA-FPFD with respect to different OOS lags are about 37 milliseconds, much less than that of the DIPDA-Re method which requires around 1040 milliseconds per scan to process the data, with the DIPDA-D method consuming the least computation time. Furthermore, in contrast to the 1000 milliseconds’ sonar sampling interval, it can be found that the proposed DIPDA-FPFD is capable of being implemented in real time while the DIPDA-Re gives delayed tracking results, and more output delay can be expected as the OOS lag increases. This is because the DIPDA-Re method needs to reprocess all the local sonars’ transmitted information to be in a chronological sequence, which consumes tremendous computation resource. As can be seen from Table 5, among the three compared algorithms, the DIPDA-Re requires the most storage memory when fusing the locally transmitted asynchronous information, followed by the proposed DIPDA-FPFD and the DIPDA-D. This is due to the fact that the DIPDA-Re needs to store all local pseudo tracks information from last fusion time till to next OOSMs coming, while the proposed DIPDA-FPFD only needs to store the state information of central tracks, whose number is much less compared to that of the local pseudo tracks. The DIPDA-D method consumes the least storage resource due to its straightforwardly discarding the OOSMs.

As a conclusion, the proposed method is able to deliver comparative tracking performance as the optimal DIPDA-Re method, while requiring much less computation and storage resources, as well as being able to be implemented in real time. Meanwhile, our proposed method gives much improved tracking performance over the low benchmark DIPDA-D method.

## 7. Conclusions

This paper proposes a novel approach for target tracking using multiple asynchronous BO sensors in the presence of clutter measurement disturbance and target misdetection. The proposed LIPDA algorithm for local measurement tracking eliminates most of false alarms and greatly improves the accuracy of the target bearing measurements. Additionally, in the fusion center, the proposed DIPDA-FPFD algorithm enables fusing the OOS information efficiently and also to operate the track management which confirms and maintains true tracks, recognizes false tracks and then deleting them from memory. The proposed methods can be directly applied in the realistic tracking applications, such as maritime surveillance, environment monitoring and autonomous driving, to name a few.

The proposed approach is able to deal with slightly maneuvering target tracking, but may deteriorate in the case of classical target maneuvering situation. This is because of the accumulated linearized errors of local measurement state model in the local tracking, and the unique motion model assumption used in the central tracking. Extending the proposed methods to deal with the classical target maneuvering problem is non-trivial but not straightforward. When incorporating the target maneuvering problem into the proposed framework, one should simultaneously consider the maneuvering impact in both the local and central tracking. A possible direction can be deriving a more complicated but accurate measurement state evolving model for the local pseudo track tracking, and deploying the interactive multiple models (IMM) or variable structure interactive multiple models (VSIMM) methods for the central tracking. Another future work will be extending the proposed methods to track multiple closely spaced targets.

## Figures and Tables

**Table 1:** Total number of initialized and transmitted pseudo tracks in sonar $s_{2}$ at case 1.

Scan Index	500	1000	1500	2000	2500
# of initialized pseudo tracks	207,144	409,344	611,521	814,362	1,020,037
# of transmitted pseudo tracks	25,297	81,277	138,342	188,992	214,774
Reduced rate (%)	87.79	80.14	77.38	76.79	78.94

**Table 2:** Total number of initialized and transmitted pseudo tracks in sonar $s_{2}$ at case 2.

Scan Index	500	1000	1500	2000	2500
# of initialized pseudo tracks	70,258	130,507	187,483	238,134	293,029
# of transmitted pseudo tracks	14,935	63,964	112,198	160,006	187,107
midrule Reduced rate (%)	78.75	50.99	40.16	32.81	36.15

**Table 3:** Total number of initialized and transmitted pseudo tracks in sonar $s_{2}$ at case 3.

Scan Index	500	1000	1500	2000	2500
# of initialized pseudo tracks	18,621	48,633	78,536	103,614	119,274
# of transmitted pseudo tracks	16,229	33,495	47,700	61,795	75,722
Reduced rate (%)	12.84	31.13	39.26	40.36	36.51

**Table 4:** Computational complexity of each compared method (take case 2 for example).

	DIPDA-FPFD			DIPDA-D	DIPDA-Re		
	1 Lag	3 Lag	10 Lag	/	1 Lag	3 Lag	10 Lag
Sonar’s sampling interval (ms)	1000						
Averaged elapsed time per scan (ms)	37.03	37.04	37.07	36.81	1037.42	1038.22	1041.02
Real time or delayed implementation	real time			real time	delayed		

**Table 5:** Storage requirement of each compared method at different scans (take case 2 for example, $l_{\max}=10$), storage requirement is evaluated by the stored number of scalars.

	Scan Index	400	1300	2100
Method				
DIPDA-FPFD		28,182	24,948	25,872
DIPDA-Re		117,214	113,118	11,554
DIPDA-D		3782	3472	3596

**Figure A1:**
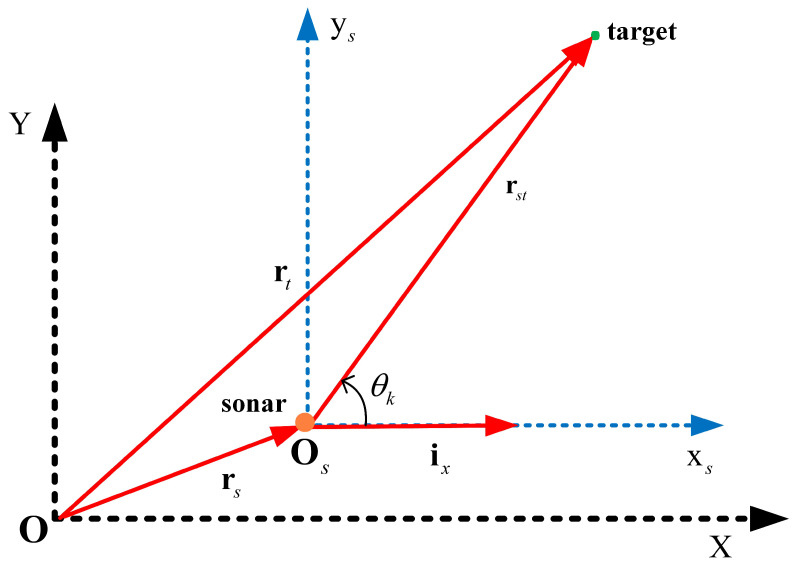
Geometry between the target and BO sensor.

## Nomenclature

The fundamental notations and acronyms used in this paper are properly defined in this section.

**A. Acronyms**	
BO	Bearing-only
OOSM	Out-of-sequence measurement
IPDA	Integrated probabilistic data association
IPDA-EKF	Integrated probabilistic data association-extended Kalman filter
LIPDA	Local integrated probabilistic data association
DIPDA-FPFD	Distributed integrated probabilistic data association-forward prediction fusion and decorrelation
SPRT	Sequential probability ratio test
PTE	Probability of target existence
LOS	Line-of-sight
RMSE	Root mean square error
DIPDA-Re	Distributed integrated probabilistic data association-reprocessing
DIPDA-D	Distributed integrated probabilistic data association-discarding
CFT	Confirmed false track
CTT	Confirmed true track
Gmix	Gaussian mixture
pdf	Probability density function
**B. Notations**	
$p_{11}$	The probability that target exists at time *k* given that it existed at time k−1
$\Delta T_{k,k-1}$	The time interval of two consecutive scans
$T_{ave}$	The averaged target existence duration
$\chi_{k}$	The event of target existence at time $t_{k}$
$x_{k}$	The target kinematic state at time $t_{k}$, with position component $x_{k}^{p}$ and velocity component $x_{k}^{v}$
$B_{k}$	The pseudo track state at time $t_{k}$
$F_{k,k-1}$	The kinematic state transition matrix from time $t_{k-1}$ to $t_{k}$
${\tilde{F}}_{k,k-1}$	The measurement state transition matrix from time $t_{k-1}$ to $t_{k}$
$w_{k}$	The process noise of target dynamic model, with zero mean and covariance $Q_{k,k-1}$
${\tilde{w}}_{k}$	The process noise of measurement state model, with zero mean and covariance ${\tilde{Q}}_{k,k-1}$
$Z_{k}^{s}$	The set of measurements received by sensor *s* at time $t_{k}$ with cardinality $M_{k}^{s}$
$Z_{k,i}^{s}$	The *i*th measurement of $Z_{k}^{s}$
$Z^{k,s}$	The set of sensor *s* received measurements up to and including time $t_{k}$
$Z^{k}$	The set of measurements collected by all sensors up to and including time $t_{k}$
$z_{k}$	The set of selected measurements at time $t_{k}$, with cardinality $m_{k}$
$z_{k,i}$	The *i*th measurement of $z_{k}$
${\hat{Z}}_{k}^{s}$	The set of refined bearing measurements of sensor *s* at time $t_{k}$
${\hat{Z}}^{k}$	The set of refined bearing measurements collected by all sensors up to and including time $t_{k}$
$P_{D}$	Target detection probability
$s_{k}$	The sensor kinematic state at time $t_{k}$, with position component $s_{k}^{p}$ and velocity component $s_{k}^{v}$
$v_{k}$	The sensor noise with zero mean and covariance $R_{k}$
$N(x;\hat{x},P)$	The Gaussian distribution of variable x with mean $\hat{x}$ and its error covariance P
$\left({\hat{x}}_{k-1|k-1},P_{k-1|k-1}\right)$	Mean and covariance of posterior kinematic state estimate at time $t_{k-1}$
$\left({\hat{x}}_{k|k-1},P_{k|k-1}\right)$	Mean and covariance of predicted kinematic state estimate at time $t_{k}$
$p_{k,i}$	The likelihood of measurement $z_{k,i}$
$P_{G}$	The probability that target measure falls into the validation gate
$\rho_{k,i}$	The clutter measurement density of $z_{k,i}$
$\beta_{k,i}$	The association probability that each measurement $z_{k,i}$ originates from the target
$\left({\hat{x}}_{k|k,i},P_{k|k,i}\right)$	Mean and covariance of kinematic state updated using $z_{k,i}$ at time $t_{k}$
$\left({\hat{B}}_{k|k},\Gamma_{k|k}\right)$	Mean and covariance of posterior local measurement state estimate at time $t_{k}$
$i_{x}$	The unit vector of the X-axis of the sonar *s* local Cartesian coordinate
$i_{s}$	The unit vector of the sonar *s* position vector in the global Cartesian coordinate
$\left(v_{\max}^{t},v_{\max}^{s}\right)$	The maximum velocity of target and sonar *s*, respectively
$\left({\bar{x}}_{k|b}^{c},{\bar{P}}_{k|b}^{c}\right)$	Mean and covariance of predicted kinematic state of central track *c* from $t_{b}$ to $t_{k}$
$\left({\hat{x}}_{\tau |b,\tau }^{c},P_{\tau |b,\tau }^{c}\right)$	Mean and covariance of track *c* kinematic state updated by ${\hat{Z}}_{\tau }^{s}$ at time $t_{\tau }$
$\left({\bar{x}}_{k|b,\tau }^{c},{\bar{P}}_{k|b,\tau }^{c}\right)$	Mean and covariance of track *c* predicted kinematic state from time $t_{\tau }$ to $t_{k}$
$\left({\hat{x}}_{k|\tau }^{c},P_{k|\tau }^{c}\right)$	Mean and covariance of track *c* kinematic state purely updated by ${\hat{Z}}_{\tau }^{s}$ at time $t_{k}$
$\left({\hat{x}}_{k|k,\tau }^{c},P_{k|k,\tau }^{c}\right)$	Mean and covariance of fused track *c* kinematic state at time $t_{k}$

## Appendix A

The geometry of the target and BO sensor in the 2D Cartesian coordinate system is depicted in Figure A1.

The Jacobian of local measurement state $B(x_{k},s_{k})$ with respect to $x_{k}$ is obtained as

(A1) $$\frac{\partial B_{k}\left(x_{k},s_{k}\right)}{\partial x_{k}}=\left[\begin{array}{cc} \frac{\partial \theta_{k}}{\partial x_{k}^{p}} & \frac{\partial \theta_{k}}{x_{k}^{v}}\\ \frac{\partial {\dot{\theta }}_{k}}{\partial x_{k}^{p}} & \frac{\partial {\dot{\theta }}_{k}}{\partial x_{k}^{v}} \end{array}\right].$$

As can be seen from Figure A1, the relative position vector between the target and sonar satisfies $r_{st}=x_{k}^{p}-s_{k}^{p}$, with $r_{s}$ and $r_{t}$ denoting the position vector of the sonar and target in the global Cartesian coordinate (XOY), respectively, and $i_{x}$ is the unit vector of the X-axis of the local sonar Cartesian coordinate ($X_{s}O_{s}Y_{s}$). The BO measurement at time *k* is defined by

(A2) $$\theta_{k}=\cos^{-1}\frac{i_{x}^{T}r_{st}}{∥i_{x}^{T}∥∥r_{st}∥}≜\cos^{-1}\left(i_{x}^{T}r_{st}\right).$$

The bearing rate ${\dot{\theta }}_{k}$ is obtained by

(A3) $$\begin{array}{c} {\dot{\theta }}_{k}=\frac{\partial \theta }{\partial t}=-\frac{1}{\sqrt{1-\cos^{2}\theta }}\frac{\partial }{\partial t}\left\{i_{x}^{T}i_{st}\right\}=-\frac{1}{\left|\sin\theta \right|}\left(\frac{\partial i_{x}^{T}}{\partial t}i_{st}+i_{x}^{T}\frac{\partial i_{st}}{\partial t}\right) \end{array},$$

where

(A4) $$\frac{\partial i_{x}^{T}}{\partial t}=0_{1\times 2},$$

and

(A5) $$\frac{\partial i_{st}}{\partial t}=\frac{\partial \frac{r_{st}}{∥r_{st}∥}}{\partial t}=\frac{\frac{\partial r_{st}}{\partial t}∥r_{st}∥-r_{st}\frac{\partial ∥r_{st}∥}{\partial t}}{{∥r_{st}∥}^{2}},$$

with $\frac{\partial r_{st}}{\partial t}$ and $\frac{\partial ∥r_{st}∥}{\partial t}$ calculated respectively by

(A6) $$\frac{\partial r_{st}}{\partial t}=\frac{\partial \left(x_{k}^{p}-s_{k}^{p}\right)}{\partial t}=x_{k}^{v}-s_{k}^{v},$$

(A7) $$\begin{array}{cc} \frac{\partial ∥r_{st}∥}{\partial t}=\frac{\partial }{\partial t}\left\{\sqrt{r_{st}^{T}r_{st}}\right\} & =\frac{1}{2}\frac{1}{∥r_{st}∥}\left\{\frac{\partial r_{st}^{T}}{\partial t}r_{st}+r_{st}^{T}\frac{\partial r_{st}}{\partial t}\right\}=\frac{1}{2∥r_{st}∥}\left\{{\left(x_{k}^{v}-s_{k}^{v}\right)}^{T}r_{st}+r_{st}^{T}\left(x_{k}^{v}-s_{k}^{v}\right)\right\}\\ & =\frac{1}{2∥r_{st}∥}\left\{r_{st}^{T}\left(x_{k}^{v}-s_{k}^{v}\right)+r_{st}^{T}\left(x_{k}^{v}-s_{k}^{v}\right)\right\}=i_{st}^{T}\left(x_{k}^{v}-s_{k}^{v}\right). \end{array}$$

Substituting Equations (A6) and (A7) into (A5), one gets

(A8) $$\frac{\partial i_{st}}{\partial t}=\frac{\left(x_{k}^{v}-s_{k}^{v}\right)∥r_{st}∥-r_{st}\left[i_{st}^{T}\left(x_{k}^{v}-s_{k}^{v}\right)\right]}{{∥r_{st}∥}^{2}}=\frac{∥r_{st}∥\left(x_{k}^{v}-s_{k}^{v}\right)-\left[i_{st}^{T}\left(x_{k}^{v}-s_{k}^{v}\right)\right]r_{st}}{{∥r_{st}∥}^{2}}.$$

Applying Equations (A4) and (A8) to (A3), one has

(A9) $$\begin{array}{cc} {\dot{\theta }}_{k} & =-\frac{1}{\left|\sin\theta \right|}\left(i_{x}^{T}\frac{∥r_{st}∥\left(x_{k}^{v}-s_{k}^{v}\right)-\left[i_{st}^{T}\left(x_{k}^{v}-s_{k}^{v}\right)\right]r_{st}}{{∥r_{st}∥}^{2}}\right)\\ & =-\frac{1}{\left|\sin\theta \right|}\left(\frac{∥r_{st}∥i_{x}^{T}\left(x_{k}^{v}-s_{k}^{v}\right)-i_{x}^{T}\left[i_{st}^{T}\left(x_{k}^{v}-s_{k}^{v}\right)\right]r_{st}}{{∥r_{st}∥}^{2}}\right)\\ & =\frac{\left[i_{st}^{T}\left(x_{k}^{v}-s_{k}^{v}\right)\right]i_{x}^{T}r_{st}-∥r_{st}∥i_{x}^{T}\left(x_{k}^{v}-s_{k}^{v}\right)}{\left|\sin\theta \right|{∥r_{st}∥}^{2}}\\ & =\frac{\left[i_{st}^{T}\left(x_{k}^{v}-s_{k}^{v}\right)\right]i_{x}^{T}i_{st}-i_{x}^{T}\left(x_{k}^{v}-s_{k}^{v}\right)}{\left|\sin\theta \right|∥r_{st}∥}\\ & =\frac{\left[i_{st}^{T}\left(x_{k}^{v}-s_{k}^{v}\right)\right]\cos\theta -i_{x}^{T}\left(x_{k}^{v}-s_{k}^{v}\right)}{\left|\sin\theta \right|∥r_{st}∥}. \end{array}$$

Based on the expression of Equations (A2) and (A9), one needs to work out each partitioned matrix in the Jacobian of local measurement state defined at (A1).

(A10) $$\frac{\partial \theta_{k}}{\partial x_{k}^{p}}=\frac{\partial }{\partial x_{k}^{p}}\left\{\cos^{-1}\left(i_{x}^{T}i_{st}\right)\right\}=-\frac{1}{\sqrt{1-\cos^{2}\theta_{k}}}\frac{\partial }{\partial x_{k}^{p}}\left(i_{x}^{T}i_{st}\right)=-\frac{1}{\left|\sin\theta_{k}\right|}\left(\frac{\partial i_{x}^{T}}{\partial x_{k}^{p}}i_{st}+i_{x}^{T}\frac{\partial i_{st}}{\partial x_{k}^{p}}\right),$$

where

(A11) $$\frac{\partial i_{x}^{T}}{\partial x_{k}^{p}}=0_{1\times 2},$$

and

(A12) $$\frac{\partial i_{st}}{\partial x_{k}^{p}}=\frac{\partial \frac{r_{st}}{∥r_{st}∥}}{\partial x_{k}^{p}}=\frac{\frac{\partial r_{st}}{\partial x_{k}^{p}}∥r_{st}∥-r_{st}\frac{\partial ∥r_{st}∥}{\partial x_{k}^{p}}}{{∥r_{st}∥}^{2}}.$$

To figure out Equation (A12), one needs to firstly calculate the $\frac{\partial r_{st}}{\partial x_{k}^{p}}$ and $\frac{\partial ∥r_{st}∥}{\partial x_{k}^{p}}$, given below,

(A13) $$\frac{\partial r_{st}}{\partial x_{k}^{p}}=\frac{\partial \left(x_{k}^{p}-s_{k}^{p}\right)}{\partial x_{k}^{p}}=\frac{\partial x_{k}^{p}}{\partial x_{k}^{p}}-\frac{\partial s_{k}^{p}}{\partial x_{k}^{p}}=I_{2}-0_{2}=I_{2},$$

(A14) $$\begin{array}{cc} \frac{\partial ∥r_{st}∥}{\partial x_{k}^{p}}=\frac{\partial }{\partial x_{k}^{p}}\left\{\sqrt{r_{st}^{T}r_{st}}\right\} & =\frac{1}{2}\frac{1}{\sqrt{r_{st}^{T}r_{st}}}\left(\frac{\partial r_{st}^{T}}{\partial x_{k}^{p}}r_{st}+r_{st}^{T}\frac{\partial r_{st}}{\partial x_{k}^{p}}\right)=\frac{1}{2}\frac{1}{∥r_{st}∥}\left(r_{st}^{T}\frac{\partial r_{st}}{\partial x_{k}^{p}}+r_{st}^{T}\frac{\partial r_{st}}{\partial x_{k}^{p}}\right)\\ & =\frac{1}{2}\frac{1}{∥r_{st}∥}\left(r_{st}^{T}I_{2}+r_{st}^{T}I_{2}\right)=\frac{1}{2∥r_{st}∥}\left(2r_{st}^{T}\right)=i_{st}^{T}. \end{array}$$

Substituting Equations (A13) and (A14) into (A12), one gets the finalized expression,

(A15) $$\frac{\partial i_{st}}{\partial x_{k}^{p}}=\frac{I_{2}∥r_{st}∥-r_{st}i_{st}^{T}}{{∥r_{st}∥}^{2}}=\frac{I_{2}-i_{st}i_{st}^{T}}{∥r_{st}∥}.$$

Finally, applying Equations (A11) and (A15) to (A10), one obtains

(A16) $$\frac{\partial \theta_{k}}{\partial x_{k}^{p}}=-\frac{1}{\left|\sin\theta \right|}\left(i_{x}^{T}\left(\frac{I_{2}-i_{st}i_{st}^{T}}{∥r_{st}∥}\right)\right)=\frac{i_{x}^{T}i_{st}i_{st}^{T}-i_{x}^{T}}{∥r_{st}∥\left|\sin\theta_{k}\right|}=\frac{i_{st}^{T}\cos\theta_{k}-i_{x}^{T}}{∥r_{st}∥\left|\sin\theta_{k}\right|}.$$

Since the bearing measurement is only the function of the target-sonar relative position, thus, one has

(A17) $$\frac{\partial \theta_{k}}{\partial x_{k}^{v}}=0_{1\times 2},$$

and

(A18) $$\frac{\partial {\dot{\theta }}_{k}}{\partial x_{k}^{v}}=\frac{\partial }{\partial x_{k}^{v}}\left\{\frac{\left[i_{st}^{T}\left(x_{k}^{v}-s_{k}^{v}\right)\right]\cos\theta -i_{x}^{T}\left(x_{k}^{v}-s_{k}^{v}\right)}{\left|\sin\theta \right|∥r_{st}∥}\right\}=\frac{i_{st}^{T}\cos\theta_{k}-i_{x}^{T}}{∥r_{st}∥\left|\sin\theta_{k}\right|},$$

with

(A19) $$\begin{array}{c} \frac{\partial {\dot{\theta }}_{k}}{\partial x_{k}^{p}}=\frac{\partial }{\partial x_{k}^{p}}\left\{\frac{\left[i_{st}^{T}\left(x_{k}^{v}-s_{k}^{v}\right)\right]\cos\theta_{k}-i_{x}^{T}\left(x_{k}^{v}-s_{k}^{v}\right)}{\left|\sin\theta_{k}\right|∥r_{st}∥}\right\}\\ =\frac{\frac{\partial }{\partial x_{k}^{p}}\left\{\left[i_{st}^{T}\left(x_{k}^{v}-s_{k}^{v}\right)\right]\cos\theta_{k}-i_{x}^{T}\left(x_{k}^{v}-s_{k}^{v}\right)\right\}\left(\left|\sin\theta_{k}\right|∥r_{st}∥\right)}{{\left(\left|\sin\theta_{k}\right|∥r_{st}∥\right)}^{2}}\\ -\frac{\left\{\left[i_{st}^{T}\left(x_{k}^{v}-s_{k}^{v}\right)\right]\cos\theta_{k}-i_{x}^{T}\left(x_{k}^{v}-s_{k}^{v}\right)\right\}\frac{\partial }{\partial x_{k}^{p}}\left\{\left|\sin\theta_{k}\right|∥r_{st}∥\right\}}{{\left(\left|\sin\theta_{k}\right|∥r_{st}∥\right)}^{2}}. \end{array}$$

After complicated mathematical transformation and simplification, the above item is eventually obtained by

(A20) $$\frac{\partial {\dot{\theta }}_{k}}{\partial x_{k}^{p}}=\left\{\begin{array}{cc} \frac{\left\{\begin{array}{c} {\left(x_{k}^{v}-s_{k}^{v}\right)}^{T}\cos\theta_{k}\sin^{2}\theta_{k}+i_{x}^{T}\left(x_{k}^{v}-s_{k}^{v}\right)i_{st}^{T}\\ +2i_{st}^{T}\left(x_{k}^{v}-s_{k}^{v}\right)\cos^{3}\theta_{k}-3i_{st}^{T}\left(x_{k}^{v}-s_{k}^{v}\right)\cos\theta_{k}i_{st}^{T}\\ +\left[i_{st}^{T}\left(x_{k}^{v}-s_{k}^{v}\right)-i_{x}^{T}\left(x_{k}^{v}-s_{k}^{v}\right)\cos\theta_{k}\right]i_{x}^{T} \end{array}\right\}}{{∥r_{st}∥}^{2}\sin^{3}\theta_{k}} & \theta_{k}\in \left(0,\pi \right)\\ \frac{\left\{\begin{array}{c} {\left(x_{k}^{v}-s_{k}^{v}\right)}^{T}\cos\theta_{k}\sin^{2}\theta_{k}+i_{x}^{T}\left(x_{k}^{v}-s_{k}^{v}\right)\cos2\theta_{k}i_{st}^{T}\\ -\left[i_{st}^{T}\left(x_{k}^{v}-s_{k}^{v}\right)\right]\cos\theta_{k}\cos2\theta_{k}i_{st}^{T}\\ +\left\{\left[i_{st}^{T}\left(x_{k}^{v}-s_{k}^{v}\right)\right]\cos2\theta_{k}-i_{x}^{T}\left(x_{k}^{v}-s_{k}^{v}\right)\cos\theta_{k}\right\}i_{x}^{T} \end{array}\right\}}{{∥r_{st}∥}^{2}\sin^{3}\theta_{k}} & \theta_{k}\in \left(\pi ,2\pi \right). \end{array}\right.$$

Maximizing the Jacobian matrix defined in Equation (A1) is equivalent to maximizing the trace of the same Jacobian matrix, then one needs to maximize the coefficient vectors of the main diagonal elements of $Q_{k,k-1}$, i.e., simultaneously maximize $\frac{\partial \theta_{k}}{\partial x_{k}^{p}}$ and $\frac{\partial {\dot{\theta }}_{k}}{\partial x_{k}^{v}}$ by adjusting the target kinematic state in the global Cartesian coordinate under the constraint that the target measurement state equals to ${\left[\theta_{k}\;{\dot{\theta }}_{k}\right]}^{T}$. One has

(A21) $$\frac{\partial \theta_{k}}{\partial x_{k}^{p}}=\frac{\partial {\dot{\theta }}_{k}}{\partial x_{k}^{v}}=\frac{i_{st}^{T}\cos\theta_{k}-i_{x}^{T}}{∥r_{st}∥\left|\sin\theta_{k}\right|}.$$

As can be seen from Figure A1, when the target lies at the opposite side of the X-axis of the local sonar Cartesian coordinate, i.e., $i_{st}=-i_{x}$, the nominator $i_{st}^{T}\cos\theta_{k}-i_{x}^{T}$ gains the maximum vector norm, besides, when the target-sonar distance equals the minimum sonar detection range, i.e., $∥r_{st}∥=r_{\min}$, the denominator $∥r_{st}∥\left|\sin\theta_{k}\right|$ gets the minimized value. As a result, when the target satisfies $i_{st}=-i_{x}$ and $∥r_{st}∥=r_{\min}$, $\frac{\partial \theta_{k}}{\partial x_{k}^{p}}$ and $\frac{\partial {\dot{\theta }}_{k}}{\partial x_{k}^{v}}$ simultaneously achieves the maximized value, and equals

(A22) $$\frac{\partial \theta_{k}}{\partial x_{k}^{p}}=\frac{\partial {\dot{\theta }}_{k}}{\partial x_{k}^{v}}=\frac{i_{x}^{T}\cos\theta_{k}-i_{x}^{T}}{r_{\min}\left|\sin\theta_{k}\right|}.$$

And also, substituting $i_{st}=-i_{x}$ and $∥r_{st}∥=r_{\min}$ into Equation (A20), one can get

(A23) $$\frac{\partial {\dot{\theta }}_{k}}{\partial x_{k}^{p}}=\left\{\begin{array}{cc} \frac{2+3\cos\theta_{k}-\cos^{3}\theta_{k}}{r_{\min}^{2}\sin^{3}\theta }\left(v_{\max}^{t}i_{x}^{T}+v_{\max}^{s}i_{s}^{T}\right) & \theta_{k}\in \left(0,\pi \right)\\ \frac{3\cos^{3}\theta_{k}+4\cos^{2}\theta_{k}-3\cos\theta_{k}-2}{r_{\min}^{2}\sin^{3}\theta_{k}}\left(v_{\max}^{t}i_{x}^{T}+v_{\max}^{s}i_{s}^{T}\right) & \theta_{k}\in \left(\pi ,2\pi \right). \end{array}\right.$$

where $v_{\max}^{t}$ and $v_{\max}^{s}$ denote the maximum velocity of target and sonar, respectively.
